# Dissecting the mediating role of cytokines in the interaction between immune traits and sepsis: insights from comprehensive mendelian randomization

**DOI:** 10.3389/fimmu.2024.1417716

**Published:** 2024-07-15

**Authors:** Xiangtao Zheng, Yihui Wang, Yuming Wang, Xiaofeng Wang, Lei Pei, Shanzhi Zhao, Fangchen Gong, Ranran Li, Huan Liu, Wenbin Liu, Enqiang Mao, Zhitao Yang, Erzhen Chen, Ying Chen

**Affiliations:** ^1^ Department of Emergency, Ruijin Hospital, Shanghai Jiao Tong University School of Medicine, Shanghai, China; ^2^ Shanghai Lung Cancer Center, Shanghai Chest Hospital, Shanghai Jiao Tong University School of Medicine, Shanghai, China; ^3^ Department of Critical Care Medicine, Ruijin Hospital, Shanghai Jiao Tong University School of Medicine, Shanghai, China

**Keywords:** cytokines, immunophenotype, mediating analysis, Mendelian randomization, sepsis

## Abstract

**Background:**

Sepsis is a life-threatening organ dysfunction resulting from a dysregulated host response to infection, yet the potential causal relationship between the immunophenotype and sepsis remains unclear.

**Methods:**

Genetic variants associated with the immunophenotype served as instrumental variables (IVs) in Mendelian randomization (MR) to elucidate the causal impact of the immunophenotype on three sepsis outcomes. Additionally, a two-step MR analysis was conducted to identify significant potential mediators between the immunophenotype and three sepsis outcomes.

**Results:**

Our MR analysis demonstrated a significant association between the immunophenotype and sepsis outcome, with 36, 36, and 45 the immunophenotype associated with the susceptibility, severity, and mortality of sepsis, respectively. Specifically, our analysis highlighted the CD14+ CD16+ monocyte phenotype as a significant factor across all three sepsis outcomes, with odds ratios (ORs) and corresponding confidence intervals (CIs) indicating its impact on sepsis (OR = 1.047, CI: 1.001-1.096), sepsis in Critical Care Units (OR = 1.139, CI: 1.014-1.279), and sepsis-related 28-day mortality (OR = 1.218, CI: 1.104-1.334). Mediation analyses identified seven cytokines as significant mediators among 91 potential cytokines, including interleukin-5 (IL-5), S100A12, TNF-related apoptosis-inducing ligand (TRAIL), T-cell surface glycoprotein CD6 isoform, cystatin D, interleukin-18 (IL-18), and urokinase-type plasminogen activator (uPA). Furthermore, reverse MR analysis revealed no causal effect of sepsis outcomes on the immunophenotype.

**Conclusion:**

Our MR study suggests that the immunophenotype is significantly associated with the susceptibility, severity, and mortality of patient with sepsis, providing, for the first time, robust evidence of significant associations between immune traits and their potential risks. This information is invaluable for clinicians and patients in making informed decisions and merits further attention.

## 1 Background

Sepsis is a life-threatening organ dysfunction caused by a dysregulated host response to infection (1). Epidemiological studies have reported a sepsis mortality rate of up to 20.6% (2, 3); however, in the event of septic shock development, the mortality rate may increase to 40%-50% (4). Initially, the immune response is characterized by excessive inflammation, which rapidly transitions to an immunosuppressive state. It is evident that immune cells play a pivotal role in the immune response and in maintaining immune homeostasis throughout sepsis. Understanding the alterations and mechanisms within diverse immune cell populations and immune traits in sepsis promises to yield novel therapeutic strategies.

Host genetic variation associated with key immune cell defects initiates the early-stage immune response in sepsis, leading to a shift towards a susceptible constitution (5). Sepsis is defined by an unbalanced immune response. Pathogens evade host defense mechanisms and release inflammatory factors, continuously stimulating and damaging host-cells, ultimately leadings to a state in which homeostasis cannot be restored. In this unbalanced response, many of the immune mechanisms that are initially activated to provide protection become harmful, are associated with excessive inflammation, and lead to immunosuppression. At the time of hospital admission, patients with sepsis exhibit signs of both inflammation and immunosuppression, involving a range of cell types and organ systems (6). The mechanisms underlying the high inflammatory state, immunosuppression, and persistent longitudinal immune system changes in critically ill patients with sepsis remain unclear. Identifying key immune cell defects or detecting immunosuppressive markers may effectively predict the onset and severity of sepsis.

Mendelian randomization represents a novel approach to causal inference, employing genetic variants, such as single nucleotide polymorphisms (SNPs), as IVs. Considering that genetic variants are randomly allocated during meiosis and are not unlikely to be influenced by external factors, they can serve as proxies for randomized groups with varying levels of exposure (7). In Mendelian randomization, genetic variants that are fixed at conception and randomly assigned to individuals serve as proxies for exposure. This method is less prone to confounding or reverse causality and enables the distinction between correlation and causation (8). Multivariable Mendelian randomization (MVMR) is an extension of the MR method, enabling an equivalent analysis of mediation within a two-step MR framework (9). As a result, causal estimates obtained from MR analyses exhibit reduced susceptibility to potential confounders and reverse causality.

In this study, we used a two-step, two-sample MR investigation to explore the mediating role of cytokines in the interaction between immune traits and sepsis, in order to improve the diagnosis and management of sepsis patients.

## 2 Methods

### 2.1 Study design

**Figure 1:**
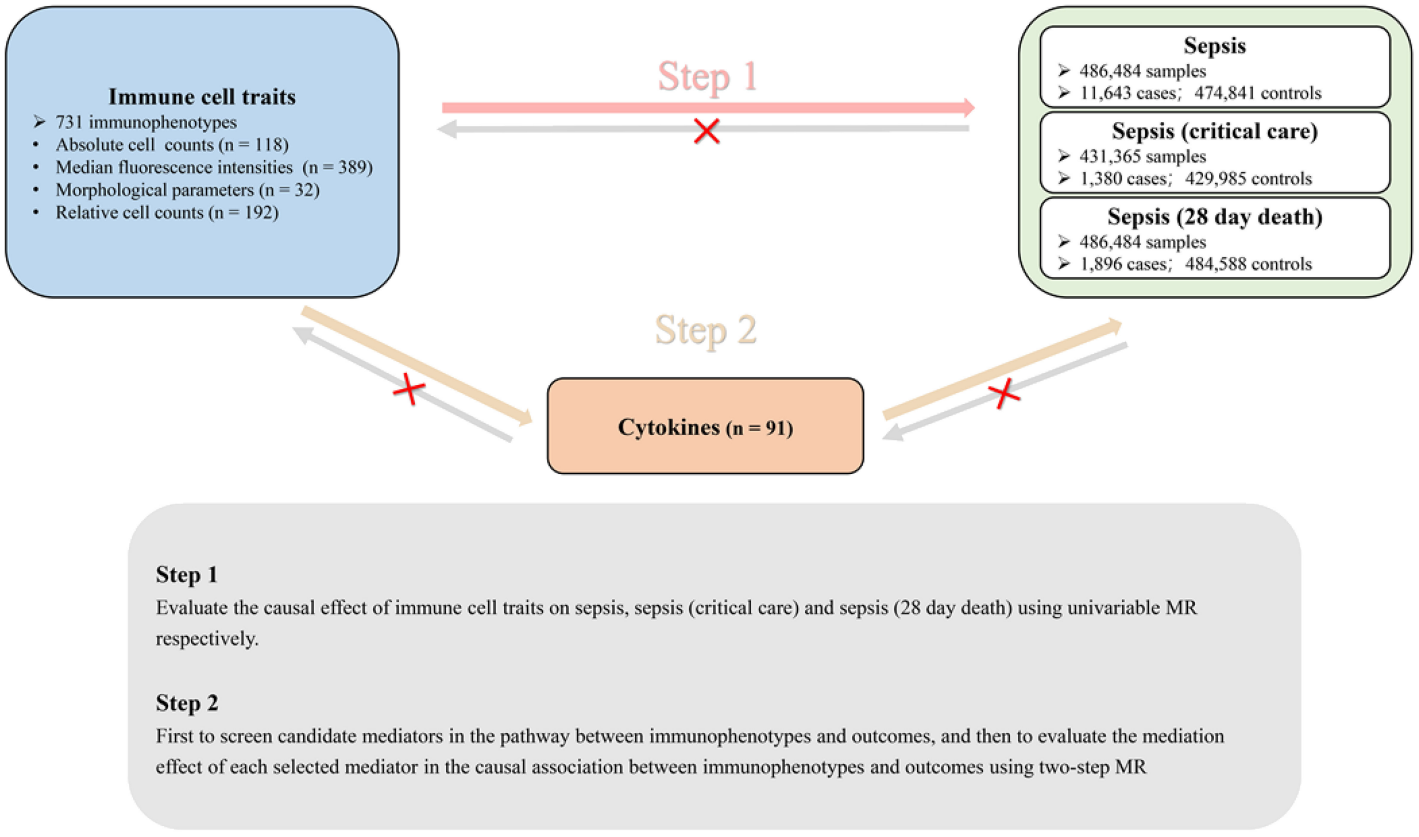
Directed acyclic graph illustrating the putative causal relationships examined in this study. This MR study comprised two analysis phases. In Phase 1, we assessed the causal associations of the immunophenotype with three septic outcomes by applying UVMR. A significant number of the immunophenotype had an independent causal effect on three septic outcomes. In Phase 2, we performed two-step MR to screen for mediators of cytokines in blood and to quantify their mediation proportions in the causal associations of the immunophenotype with three septic outcomes. Additionally, reverse MR was performed to identify the direct and independent casual effect of the immunophenotype and sepsis outcomes.

We conducted a two-step Mendelian randomization (TS-MR) study with two analysis phases to explore the causal pathway from immune cell traits to sepsis, as depicted in **Figure 1**. In Phase 1, we assessed the causal associations between immune cell traits and sepsis, severe sepsis, and sepsis-related mortality using univariable MR (UVMR), revealing an independent causal effect of immune cell traits on sepsis. In Phase 2, we identified 91 cytokines as candidate mediators involved in the causal associations between immune cell traits and sepsis. Subsequently, we quantified their mediating effects using a TS-MR approach. Additionally, reverse MR analyses were used to validate the causal effect of immune cell traits on sepsis. Our study adhered to the Strengthening the Reporting of Observational Studies in Epidemiology (STROBE-MR) guidelines (10).

### 2.2 Data sources for exposures, mediators, and outcomes

Exposure, mediator, and outcome data were obtained from summary-level statistics of GWASs conducted in individuals of European descent, sourced from reputable consortia or studies (**Table 1**).

**Table 1:** Summary information for the genetic data used in the present study.

phenotype	GWAS	population	sample size
Sepsis	UK Biobank	European	486,484
Sepsis (Critical Care Units)	UK Biobank	European	431,365
Sepsis (28-day death)	UK Biobank	European	486,484
Immune traits	GCST90001391- GCST90002121 (11)	European	3,757

#### 2.2.1 Exposures

The GWAS summary statistics for each immune trait are accessible from the GWAS Catalogue (accession numbers from GCST0001391 to GCST0002121) (11). The study included a total of 731 the immunophenotype, encompassing absolute cell (AC) counts (n = 118), median fluorescence intensities (MFIs) reflecting surface antigen levels (n = 389), morphological parameters (MPs) (n = 32), and relative cell (RC) counts (n = 192). Specifically, the features measured included MFI, AC, and RC, comprising B-cells, CDCs, mature stages of T-cells, monocytes, myeloid cells, TBNK (T-cells, B-cells, natural killer cells), and Treg panels, whereas the MP feature encompassed CDC and TBNK panels. The original GWAS on immune traits was performed using data from 3,757 individuals of European descent and there were no overlapping cohorts. For the main MR analysis, we included independent SNPs (r2< 0.001 within 10,000-kb windows), strongly associated (*P* ≤ 1E−05) with each immune cell traits.

#### 2.2.2 Mediators

The GWAS summary statistics for each plasma pro are publicly available from the GWAS Catalogue (accession numbers from GCST90274758 to GCST90274848) (12). A genome-wide protein quantitative trait locus (pQTL) study was conducted on 91 plasma proteins using the Olink Target platform in 14,824 participants.

#### 2.2.3 Outcomes

Summary-level GWAS data for sepsis were sourced from the most recent GWAS conducted by the UK Biobank, involving a total of 486,484 individuals of European descent. Summary-level GWAS data for sepsis (Critical Care Units) were obtained from the latest GWAS conducted by the UK Biobank, encompassing a total of 431,365 individuals of European descent. Summary-level GWAS data for sepsis (28-day mortality) were obtained from the latest GWAS conducted by the UK Biobank, encompassing a total of 486,484 individuals of European descent. For sepsis related MR analysis, we selected all single nucleotide polymorphisms (SNPs) associated with the exposure of interest at a genome-wide significance threshold (*P*< 5 × 10^−8^) as instrumental variables.

### 2.3 Mediation MR analysis

A Two-step MR analysis was conducted to investigate the potential mediating role of an intermediate factor between immune cell traits and sepsis. In the initial step, we determined the causal effect of genetically determined immune cell traits on the mediator (β1) using UVMR. The second step was to estimate the causal effect of each mediator on outcomes (β2), which is based on the premise that the mediator is causally associated with the outcomes of UVMR. The mediation proportion of each mediator in the associations between immune cell traits and sepsis was calculated as the product of β1 and β2 divided by the total effect of immune cell traits on the outcomes.

### 2.4 MR sensitivity analysis

In the UVMR analysis, the weighted median, weighted mode, and MR-Egger were used to confirm the robustness of the inverse variance weighting (IVW) results under various assumptions. The MR-Egger method identifies bias due to directional pleiotropy based on its intercept term, considering deviation from zero (*P* for Egger intercept<0.05) as indicative of directional pleiotropic bias (13). The F statistic was used to evaluate the validity of the IVs, and Cochran’s Q was used to assess the heterogeneity among the IVs.

### 2.5 Statistical analysis

All analyses in this study utilized the R packages TwoSampleMR (version 0.5.6), MVMR (version 0.3) and MRPRESSO (version 1.0) in R software (version 4.0.3; R Development Core Team). A *P* value<0.05 indicated statistical significance. For each SNP, the F statistic was calculated using the formula $\text{F}=\frac{R^{2}\times \left(N-2\right)}{1-R^{2}}$ where $R^{2}=\frac{2\times \beta^{2}\times EAF\times \left(1-EAF\right)}{2\times \beta^{2}\times EAF\times \left(1-EAF\right)+2\times SE^{2}\times N\times EAF\times \left(1-EAF\right)}$ , N represents the number of participants, EAF represents the effect allele frequency, and β is the estimated effect of the SNP to assess its ability to uniquely predict the outcome (14–16). The IVW estimates were considered causal associations only if they had the same direction and statistical significance as at least one sensitivity analysis and had no evidence of pleiotropy (*P* for Egger intercept >0.05). The MR estimates are presented as ORs, β coefficients or proportions, with corresponding 95% CIs.

## 3 Results

### 3.1 Basic characteristics of the MR study

An overview of the study design is shown in **Figure 1**. Details regarding genome-wide association study (GWAS) datasets for exposure, mediator, and study outcomes can be found in **Table 1**.

### 3.2 Total effect of the immunophenotype on risk of sepsis, sepsis (critical care units) and sepsis (28-day death)

**Figure 2:**
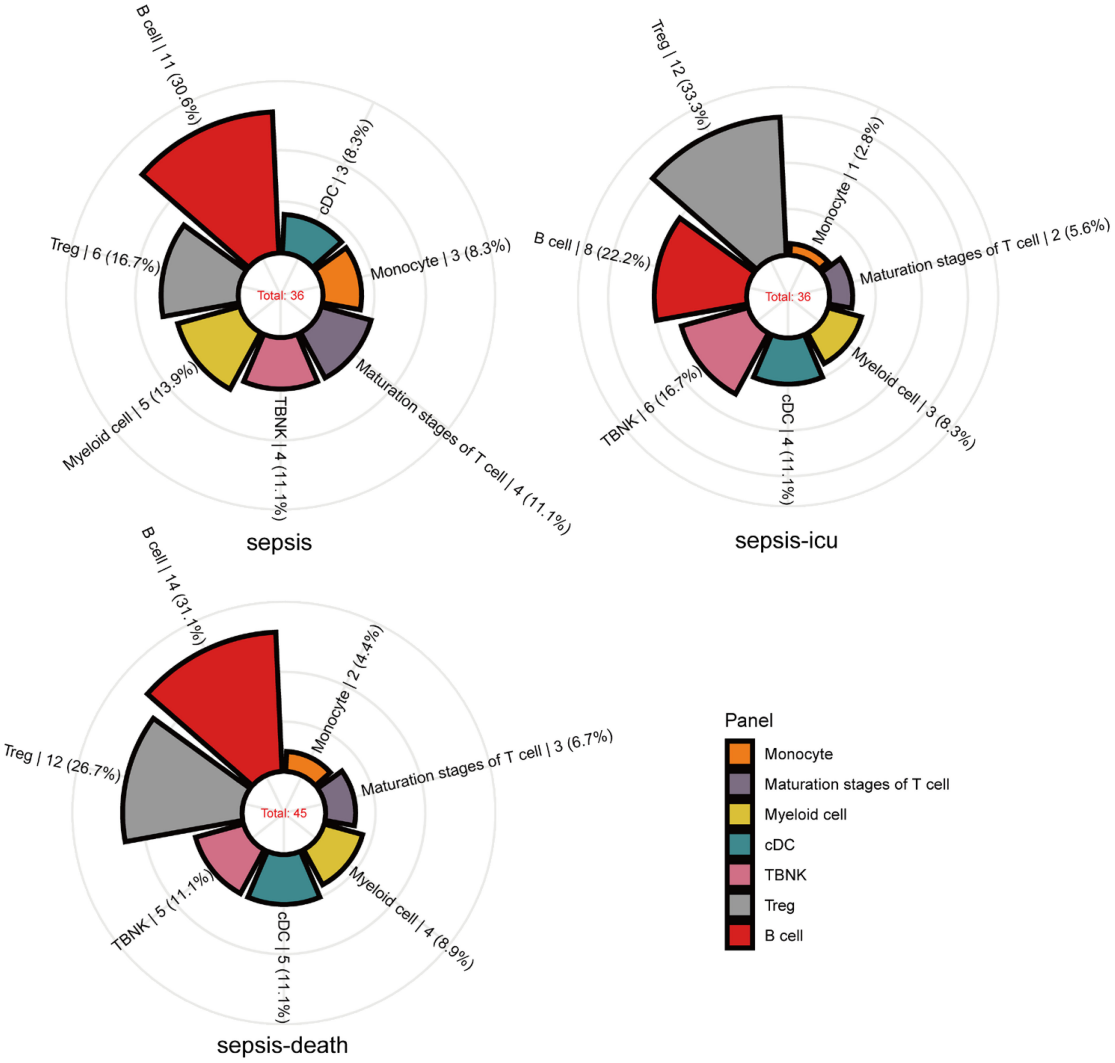
Mendelian randomization associations of immune traits with sepsis (susceptibility, severity and mortality) derived from IVW analysis. Mendelian randomization associations of the immunophenotype (seven panels) with sepsis outcomes derived from the IVW analysis. The immune traits positively associated with sepsis outcomes are shown in the rose diagram.

Our research identified significant causal relationships between various immune cells and the risk of sepsis outcomes. Initially, we found that alterations in the levels of 36 immune cells are linked to sepsis susceptibility. Specifically, an increase in 15 types and a decrease in 21 types of immune cells were observed to influence sepsis risk. These cells are categorized as follows: 11 B-cells, 3 conventional dendritic cells (cDCs), 4 in T-cell maturation stages, 3 monocytes, 5 myeloid cells, 4 TBNK cells, and 6 in T regulatory (Treg) panels (**Figure 2**). In a closely related study focusing on severe sepsis, we observed a similar pattern with a slightly altered distribution among the same 36 immune cells, where 16 showed increased levels and 20 showed decreased levels associated with sepsis risk. There were 8 B-cells, 4 cDCs, 2 in the T-cell maturation stages, 1 monocyte, 3 myeloid cells, 6 TBNK cells, and 12 in the Treg panels (see **Figure 2**). Expanding our analysis to the relationship between immune cell levels and sepsis-related 28-day mortality revealed causal links involving 45 immune cells. Here, the presence of increased levels of 17 immune cells and decreased levels of 28 immune cells were indicative of increased sepsis risk. The breakdown included 14 B-cells, 5 cDCs, 3 in the T-cell maturation stage, 2 monocytes, 4 myeloid cells, 5 TBNK cells, and 12 in the Treg panels (as shown in **Figure 2**).

**Supplementary Figures S3**, **S4**, and **S5** show varying levels of suggestive associations (p< 0.05) between different immune cell pairs and sepsis risk, as determined by Inverse variance weighted MR analysis. Specifically, **Supplementary Figure S3** identifies significant pairings involving 7, 11, 17, and 1 instance for AC, RC, MFI, and MP, respectively, in the context of sepsis. **Supplementary Figure S4** extends this analysis to Critical Care Units scenarios, highlighting 7, 8, 18, and 3 significant pairings for the same immune cell categories. **Supplementary Figure S5** further illustrates 4, 14, 23, and 4 significant associations with 28 sepsis-related deaths. Across these outcomes, the T-cell (including Treg and TBNK) and B-cell panels emerged as the most significantly associated with sepsis, surpassing other immune cell groups in the number of suggestive associations. No heterogeneity among instrumental variables was observed (all *P*-values for heterogeneity tests ≥0.05) and the few horizontal pleiotropies observed were excluded from further analysis (**Supplementary Tables 1**-**3**). In order to control the false positive rate of multiple hypothesis testing, we correct for multiple testing in this exploratory study using Benjamini-Hochberg method (17). After stratified FDR correction, the significance of immunophenotype results was not remained (all *P*-values for FDR tests > 10%, **Supplementary Tables 4**-**6**), which means that there is a potential correlation between the immunophenotypes and sepsis outcomes.

**Figure 3:**
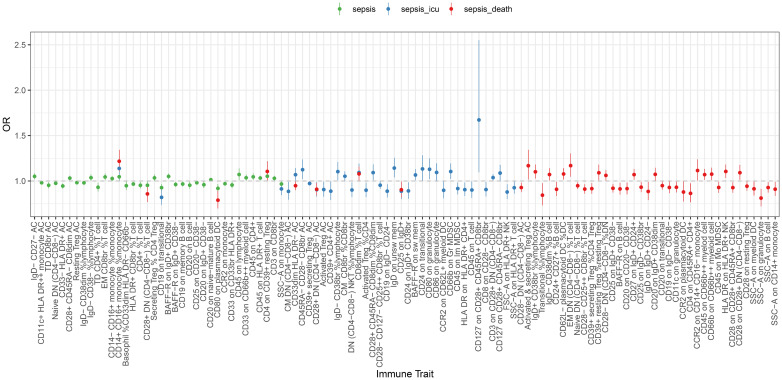
Forest plot of causal effects of sepsis (susceptibility, severity and mortality) on different immune traits. Two-step MR was used to evaluate the mediating role of each mediator in the causal associations of the immunophenotype with sepsis outcomes. MR estimates were derived from the IVW method in UVMR. All the statistical tests were two-sided. P< 0.05 was considered significant.

In our analysis of immune cell panels with significant associations with sepsis outcomes, we observed that some panels showed significant associations across various stages of the disease. In particular, the relative count of CD14+ CD16+ monocytes, expressed as a percentage of the total monocyte population, increased from general sepsis to Critical Care Units sepsis and finally to sepsis, culminating in 28-day mortality (sepsis: OR = 1.047, CI: 1-1.1.096; *P* = 0.048; sepsis of Critical Care Units: OR = 1.139, CI: 1.014-1.1.279; *P* = 0.028; sepsis of 28-day death: OR = 1.218, CI: 1.104-1.1.334; *P*< 0.001; **Figure 3**; **Supplementary Figures 3**-**5**). These findings underscore the pivotal role of CD14+ CD16+ monocytes in the progression of sepsis.

### 3.3 Reverse MR of the immunophenotype and septic outcomes

In reverse UVMR, we observed significant causal effects of sepsis, sepsis related to Critical Care Units and sepsis related to 28-day mortality on immune cell traits (**Supplementary Figures 1**, **2**). Notably, the the immunophenotype identified as significant in the reverse MR analysis differed entirely from those reported in the previous MR results, as detailed in **Figure 3**.

### 3.4 Mediation analysis of immune traits on sepsis outcomes

It is well known that cytokines have complex interaction patterns that mediate the function of immune cells. We attempted to test the potential effects of the 91 cytokines on mediating the associations between immune traits (with nominal significance) and three sepsis outcomes. As expected, our analysis revealed a significant link between the MFI of BAFF-R on IgD+ CD38br B-cells and sepsis, with interleukin-5 (IL-5) acting as a mediator. Furthermore, we identified significant associations in severe sepsis: the absolute count of CD45RA- CD28 CD8br Treg cells and the morphological parameter SSC-A on HLA DR+ T-cells were associated with severe sepsis through the mediation of S100A12 protein levels and TNF-related apoptosis-inducing ligand (TRAIL), respectively. In the context of sepsis leading to 28-day mortality, significant associations were found for the relative count of CD28+ DN Treg cells within T-cells, the morphological parameter SSC-A on B-cells, the MFI of HLA DR on HLA DR+ NK cells, and CD25 on IgD+ B-cells, which are mediated by the levels of the T-cell surface glycoprotein CD6 isoform, cystatin D, interleukin-18 (IL-18), and urokinase-type plasminogen activator (uPA), respectively (**Table 2**). Cytokines including IL-5 (mediating proportion, 7.51%), S100A12 (8.77%), TRAIL (7.46%), T-cell surface glycoprotein CD6 isoform (3.88%), cystatin D (3.34%), IL-18 (5.99%) and uPA (5.57%) each mediated the total effect of the immunophenotype on septic outcomes (**Table 2**).

**Table 2:** Mediation analysis of immune traits on sepsis (Susceptibility, Severity and mortality).

Panel	Immunophenotype	Cytokines trait	b1	se1	b2	se2	b	se	Mediating effect
B-cell	BAFF-R on IgD+ CD38br	Interleukin-5 levels	0.033737	0.013739	0.110456	0.046871	0.04959	0.015314	7.51%
Treg	CD45RA- CD28- CD8br AC	Protein S100-A12 levels	-0.03639	0.014496	-0.28403	0.144604	0.117863	0.048863	8.77%
TBNK	SSC-A on HLA DR+ T-cell	TNF-related apoptosis-inducing ligand levels	-0.03013	0.011095	0.190397	0.080206	-0.07688	0.036912	7.46%
Treg	CD28+ DN (CD4-CD8-) %T-cell	T-cell surface glycoprotein CD6 isoform levels	0.035021	0.017595	-0.16941	0.059761	-0.15308	0.05637	3.88%
TBNK	SSC-A on B-cell	Cystatin D levels	0.021814	0.010269	-0.11516	0.058254	-0.07521	0.032274	3.34%
TBNK	HLA DR on HLA DR+ NK	Interleukin-18 levels	-0.03244	0.012735	-0.18558	0.08497	0.100434	0.034101	5.99%
B-cell	CD25 on IgD+	Urokinase-type plasminogen activator levels	-0.03625	0.017712	0.154917	0.076932	-0.10077	0.048974	5.57%

b1: Directed effect of exposure on mediator; b2: Directed effect of mediator on outcome; b: Total effect of exposure on outcome.

se1: Standard error of exposure on mediator; se2: Standard error of mediator on outcome; se: Standard error of exposure on outcom.blue: sepsis outcome; yellow: sepsis outcome (Critical Care Units); green: sepsis outcome (28-day death).

## 4 Discussion

This Mendelian randomization study elucidates the causal relationships between the immunophenotype and various aspects of sepsis, including susceptibility, severity, and mortality, with a particular focus on the intermediary roles of cytokines. Through a comprehensive evaluation of 91 cytokines as potential mediators, we pinpointed those that significantly influence the pathways linking the immunophenotype to three sepsis-related outcomes. Our findings indicate that 36 the immunophenotype are associated with sepsis susceptibility, another set of 36 are associated with sepsis severity, and 45 with mortality risk. Furthermore, we identified seven cytokines that significantly mediate the causal effects of the immunophenotype on these sepsis outcomes.

At a nominal significance level, our analysis revealed causal implications of 36, 36, and 45 immune cells in the susceptibility, severity, and mortality of sepsis, respectively.

Within the significant the immunophenotype identified, the T-cell panel was predominant, comprising approximately 45% of the total, followed by the B-cell panel at 29%, and both the cDC and myeloid cell panels at 10.5% each, with the monocyte panel making up 5%. This distribution highlights the integral role of immune cells, particularly those involved in active immunity such as T-cells and B-cells. T-cells, key players in adaptive immunity, are significantly linked to sepsis-induced immunosuppression (18).

In the context of sepsis, significant alterations in T-cell metabolism occur, leading to the apoptosis of these cells. This apoptosis, particularly of CD4+ and CD8+ T-cells, results in a decrease in lymphocyte counts in late-stage sepsis patients, contributing to immunosuppression and a heightened risk of secondary infections (19). According to our MR analysis, Tregs constitute 58% of the T-cell population, underscoring their critical role in the progression of sepsis. Tregs, known for their immunoregulatory capabilities, can suppress the proinflammatory responses of effector T-cells (20), thereby playing a pivotal role in sepsis-induced immune dysregulation and increasing mortality rates among sepsis patients (21). Within the B-cell compartment, diverse functionalities are observed, with the distribution of B-cell subsets undergoing significant changes during sepsis. Memory B-cells showed more apoptosis than other types of B-cells. Infection also causes a continuous decrease in primitive B-cells and an increase in B-cell exhaustion, suggesting that the function of B-cells involved in the acquired immune response is impaired (22). Therefore, the dynamics of both T and B-cells are intricately linked to the pathophysiology of sepsis, serving as potential markers for sepsis progression and the risk associated with sepsis severity.

Our MR analysis revealed that the proportion of CD14+ CD16+ monocytes relative to the total monocyte population played a pivotal role in all three sepsis outcomes, demonstrating a direct correlation with disease severity. This finding suggested that an increased proportion of CD14+ CD16+ monocytes is associated with increased sepsis severity. In contemporary research, circulating human monocytes are categorized into subpopulations based on the expression of the surface markers CD14 (a cell coreceptor for lipopolysaccharide [LPS]) and CD16 (a low-affinity IgG receptor). These subpopulations are primarily divided into three subsets: approximately 90% are classified as classical monocytes (CD14hiCD16-), and the remaining 10% are divided into two groups: (a) intermediate monocytes (CD14hi CD16+), marked by high CD14 and low CD16 expression, and (b) nonclassical monocytes (CD14-/lo CD16+), distinguished by lower CD14 and higher CD16 expression (23–25).. CD14, a surface glycoprotein, is integral to the family of Toll-like receptors and is predominantly expressed on macrophages and monocytes, demonstrating a particular affinity for bacterial components such as lipopolysaccharides (26). Monocytes expressing CD16 are recognized for their substantial production of proinflammatory cytokines, including TNF-α and IL-1β, which are key players in inflammatory responses (27–29). These CD16+ monocytes play distinctive roles in processes such as angiogenesis, generation of reactive oxygen species, and patrolling functions (27, 30–32). Compared to conventional CD14+ CD16- monocytes, CD16+ monocytes display traits akin to those of inflammatory tissue macrophages, notably through elevated expression of MHC class II antigens and various adhesion molecules, coupled with a diminished production of the anti-inflammatory cytokine IL-10. Overall, our research demonstrated a significant correlation between the prevalence of CD14+ CD16+ monocytes and the severity of sepsis, suggesting that their increased presence may serve as an indicator of a more severe manifestation of the condition.

As outlined in **Table 2**, our analysis identified seven significant mediators that influence the overall impact of the immunophenotype on sepsis outcomes. Based on the significant results of the previous MR analysis, the mediating role of inflammatory factors between immune cell phenotype and sepsis outcome was further evaluated by mediation analysis, and finally 7 meaningful combinations were screened out. Notably, the interaction between BAFF-R on IgD+ CD38br B-cells and IL-5 was found to elevate IL-5 levels, indirectly increasing sepsis susceptibility. IL-5 is known for its role as a growth factor for eosinophils and B-cells. Mouse IL-5 promotes antigen-stimulated B-cells to differentiate into antibody-producing cells and the stimulating effect of IL-5 is similar to that of IL-6 (33–36). This similarity might contribute to the observed significant correlation between the MFI of BAFF-R on IgD+ CD38br B-cells and sepsis, as mediated by IL-5 levels. Furthermore, our mediating analysis revealed that effector memory T-cells (CD45RA- CD28- CD8br) could reduce the protein levels of S100A12, thereby exacerbating the severity of sepsis. S100A12 is mainly expressed in macrophages, which is highly correlated with inflammation and can be specifically secreted to the inflammatory site, causing the secretion of inflammatory mediators by interacting with RAGE (37). A recent research demonstrated that human S100A12 was an endogenous TLR4 ligand that induces monocyte activation, thereby acting as an amplifier of innate immunity during early inflammation and the development of sepsis (38). Those with a CD28−CD45RA− phenotype are intermediate between memory and effector CD8+ T cells (memory/effector CD8+ T cells) (39). However, the relationship between CD45RA- CD28- CD8br T cells and S100A12 has not been elucidated in the existing research, so it is difficult to directly explain the results of MR analysis based on the existing research. The relationship between memory T cells and S100A12 needs to be further studied and explored through subsequent basic research. Furthermore, our findings indicate that the morphological parameter SSC-A on HLA DR+ T-cells can decrease TRAIL levels. HLA-DR, a key class II major histocompatibility complex (MHC) antigen in humans is primarily expressed on B lymphocytes, monocytes, and macrophages and plays a crucial role in antigen presentation to CD4+ T-cells. While most T-cells typically lack HLA-DR expression, it can be induced in activated T-cells during the late stages of an immune response (40, 41), meaning that HLA-DR has high sensitivity and specificity for bacterial infection and has important value for the early diagnosis of infection (42). In the context of sepsis leading to 28-day mortality, our analysis showed that the relative count of CD28+ DN Treg cells within the T-cell population could increase the levels of the T-cell surface glycoprotein CD6 isoform. Double negative (DN) T cells express CD3 positive but lack CD4 and CD8 co-receptors, generally accounting for 1-3% of peripheral blood. DN T cells have congenital and adaptive immune functions, and have multiple effector functions, which are different from traditional CD4 + and CD8 + T cells. For example, as a T regulatory cell (Treg) function, DN T cells can inhibit GVHD and have therapeutic value for autoimmune diseases (43). CD28 is a costimulatory receptor, which plays an important role in T cell activation. In the absence of CD28 signal transduction, T cells enter an incompetent state. In this state, T cells lose further response to antigens, and the function of immune response will be limited. Decreased expression of CD28 is considered to be a marker of T cell senescence, while CD28 + Treg cells enhance the role of immunosuppression, which may be the cause of increased risk of sepsis death (44, 45). CD6 is able to impact on the efficiency of Tregs, and when CD6 expression is increased directly or indirectly, Tregs are more able to repress immune responses, which may aggravate sepsis (46). By and Large, increasing account of CD28+ DN Treg cells may increase the levels of the T-cell surface glycoprotein CD6 isoform and enhance the efficiency of Tregs to repress immune responses, aggravating sepsis. Similarly, the morphological parameter SSC-A on B-cells was associated with increased levels of cystatin D, which also contributes to deceased sepsis mortality. There are few studies on the elaboration and role of cystatin D, and no relevant studies have reported the relationship among B cells, cystatin D and sepsis. The relationship between the three needs to be further explored through basic experiments. HLA-DR on HLA-DR+ NK cells can increase the level of IL-18, aggravating the mortality of sepsis patients. HLA-DR is a common alternative to lymphocyte immune activation. Some research found that HLA-DR+NK cells possessed better capacity to produce IFN-γ in response to cytokine stimulation compared to their HLA-DR-counterparts (47). IL-18 can cause the induction of HLA-DR expression *de novo* and/or active proliferation of already existing HLA-DR+ NK cell subset (48), which may increase the production of other cytokines such as IL-18, aggravating inflammatory response. Additionally, CD25 expression on IgD+ B-cells was found to decrease uPA levels, further alleviating sepsis mortality. As an immunoregulatory cell, CD25 + B cells can secrete anti-inflammatory factors such as IL-10 and TGF-β to participate in immune regulation (49). Interleukin-2 receptor-α (IL-2Rα, CD25) is upregulated on macrophages and lymphocytes by cytokines, such as interferon-γ, interleukin-2 and interleukin-3, or by nonspecific stimulation of human monocytes with lipopolysaccharides (50, 51). In addition, Falkenberg et.al (52) found that all sections negative for CD25 were also negative for u-PA, and all sections positive for CD25 were also positive for u-PA in macrophages, but there are no relative research investigating the relationship of CD25+ B cell and u-PA, which need more basic researches to find the potential relationship between CD25+ B cell and u-PA. In conclusion, the results of mediation analysis provide us with some new directions to explore the potential relationship between inflammatory factors in immune cells and sepsis outcomes. Although there are not many related studies, it is worth further exploring in the future.

This study delineates the causal relationships between the immunophenotype and three distinct sepsis outcomes, providing an in-depth analysis of the mediators involved, particularly cytokines. The study’s key strengths include: (1) a rigorous MR study design with normative and specific criteria for mediator screening; (2) substantial sample sizes in each GWAS ensuring ample statistical power; (3) robust causal associations identified through the main analysis and supported by various sensitivity analyses; and (4) the introduction of novel insights into the field. However, there are several limitations to consider: (1) The lack of individual-level data precludes a thorough assessment of selection bias associated with binary exposures; (2) the selection bias caused by binary exposure cannot be properly evaluated since individual-level data are unavailable; and (3) our findings were derived from GWASs predominantly conducted in individuals of European ancestry and generalizing our results to other ethnic groups requires further investigation. Therefore, future studies should incorporate a clinical equivalence framework to explore the immunophenotype more extensively.

## 5 Conclusion

Our MR study demonstrated a significant association between the immunophenotype and the susceptibility, severity, and mortality of patients with sepsis, providing, for the first time, substantial evidence of the role of CD14+ CD16+ monocytes in the potential sepsis-related risks. This novel insight is crucial for clinicians and patients alike, emphasizing the need for increased awareness and consideration in clinical decision-making processes.

## Data availability statement

Data are available in a public, open access repository. Data URLs: GWAS summary statistics for 731 immune traits could be download form GWAS Catalog (Study accession: GCST90001391 (https://www.ebi.ac.uk/gwas/studies/GCST90001391) to GCST90002121 (https://www.ebi.ac.uk/gwas/studies/GCST90002121). GWAS summary statistics for sepsis outcomes could be available form https://gwas.mrcieu.ac.uk/. All codes used in the research are available from the corresponding authors.

## Author contributions

XZ: Conceptualization, Methodology, Validation, Visualization, Writing – original draft, Writing – review & editing. YHW: Writing – original draft, Writing – review & editing, Supervision. YMW: Conceptualization, Methodology, Writing – review & editing. XW: Methodology, Writing – review & editing. LP: Supervision, Writing – review & editing. SZ: Conceptualization, Writing – review & editing. FG: Supervision, Writing – review & editing. RL: Supervision, Writing – review & editing. HL: Methodology, Writing – review & editing. WL: Writing – review & editing. EM: Supervision, Writing – review & editing. ZY: Validation, Writing – review & editing. EC: Supervision, Writing – original draft, Writing – review & editing. YC: Conceptualization, Writing – original draft, Writing – review & editing.
