# Supplementary material for: Dissecting the mediating role of cytokines in the interaction between immune traits and sepsis: insights from comprehensive mendelian randomization
Source: Front Immunol. 2024 Jul 15;15:1417716. doi: 10.3389/fimmu.2024.1417716 (PMC11284126; doi:10.3389/fimmu.2024.1417716)
Supplement: Supplementary file 1 [file DataSheet_1.docx]

**Supplementary Table 1. UVMR directional pleiotropy test and heterogeneity test for the causal associations of immunophenotypes and sepsis.**

| **Exposure** | **Traits** | **Outcome** | **Directional pleiotropy test** | | | **Heterogeneity test** | | |
| --- | --- | --- | --- | --- | --- | --- | --- | --- |
|  |  |  | **Egger intercept** | **SE** | **P value** | **Q statistic** | **Q df** | **Q p value** |
| GCST90001529 | CD33- HLA DR+ AC | sepsis | -0.006747298 | 0.0076668 | 0.39185 | 17.44581165 | 17 | 0.42457971 |
| GCST90002029 | CD19 on transitional | sepsis | -0.000810136 | 0.0102504 | 0.937719 | 28.56974004 | 23 | 0.19500075 |
| GCST90001713 | CD80 on plasmacytoid DC | sepsis | 0.014780968 | 0.0106128 | 0.187058 | 12.88439591 | 14 | 0.535646 |
| GCST90001472 | BAFF-R on IgD+ CD38br | sepsis | -0.01280946 | 0.0089205 | 0.16916 | 21.89706489 | 18 | 0.23658721 |
| GCST90001473 | IgD- CD27- AC | sepsis | -0.018039601 | 0.0074964 | 0.027067 | 24.57422217 | 19 | 0.17503955 |
| GCST90001489 | CCR2 on monocyte | sepsis | -0.010132241 | 0.0053358 | 0.068309 | 28.65750366 | 28 | 0.43004975 |
| GCST90001970 | HLA DR+ CD8br %T cell | sepsis | -0.012417754 | 0.0076343 | 0.120297 | 25.82507016 | 20 | 0.17166732 |
| GCST90001474 | BAFF-R on IgD+ CD38- | sepsis | -0.002477152 | 0.0064084 | 0.702978 | 25.420277 | 22 | 0.27740191 |
| GCST90001922 | TD CD4+ %T cell | sepsis | -0.017492572 | 0.0147985 | 0.255587 | 12.13389908 | 16 | 0.73471208 |
| GCST90001585 | TD CD8br AC | sepsis | -0.003958623 | 0.0094756 | 0.680163 | 13.71884868 | 23 | 0.93464042 |
| GCST90001580 | CD4+ %T cell | sepsis | -0.000999146 | 0.0080573 | 0.902685 | 15.42684563 | 19 | 0.69510957 |
| GCST90001866 | CD4 on CD39+ resting Treg | sepsis | 0.005792415 | 0.0142236 | 0.691645 | 5.257281825 | 12 | 0.94882874 |
| GCST90001556 | SSC-A on monocyte | sepsis | -0.005145992 | 0.0081704 | 0.541665 | 6.948937782 | 12 | 0.86096942 |
| GCST90001468 | CD45 on CD4+ | sepsis | -0.016414355 | 0.0092647 | 0.099865 | 12.22940399 | 14 | 0.58788566 |
| GCST90001495 | Naive DN (CD4-CD8-) AC | sepsis | -0.008912836 | 0.0069398 | 0.211823 | 20.00122639 | 24 | 0.6967064 |
| GCST90001519 | CD45 on HLA DR+ T cell | sepsis | -0.0049522 | 0.0074976 | 0.517781 | 10.03557239 | 18 | 0.93073912 |
| GCST90002046 | CD11c+ HLA DR++ monocyte AC | sepsis | -0.007418021 | 0.0057941 | 0.215098 | 20.52191952 | 21 | 0.48845162 |
| GCST90001863 | EM CD8br %T cell | sepsis | 0.001771686 | 0.0105526 | 0.868651 | 12.73025965 | 18 | 0.80734178 |
| GCST90002005 | CD33 on CD33br HLA DR+ | sepsis | 0.00089562 | 0.0092418 | 0.923813 | 24.46816034 | 20 | 0.22253982 |
| GCST90001923 | CD19 on memory B cell | sepsis | -0.009812031 | 0.0062617 | 0.137964 | 9.098152138 | 16 | 0.90931644 |
| GCST90001781 | CD28+ CD45RA- CD8dim AC | sepsis | 0.007437149 | 0.0071895 | 0.312168 | 22.28405986 | 23 | 0.503163 |
| GCST90002031 | IgD- CD38dim %lymphocyte | sepsis | -0.007199072 | 0.0053535 | 0.193055 | 20.46641618 | 22 | 0.5538892 |
| GCST90001601 | CD20 on B cell | sepsis | 0.009353755 | 0.0077703 | 0.245167 | 22.23824057 | 18 | 0.22157959 |
| GCST90001491 | CD25 on CD20- CD38- | sepsis | -0.005829609 | 0.0058687 | 0.330456 | 20.10419794 | 25 | 0.74133829 |
| GCST90001691 | Resting Treg AC | sepsis | 0.005659597 | 0.0049341 | 0.263159 | 23.01198067 | 24 | 0.51909092 |
| GCST90001770 | CD45 on lymphocyte | sepsis | -0.019112857 | 0.0110583 | 0.144494 | 8.417895391 | 6 | 0.20905733 |
| GCST90001652 | CD20 on IgD+ CD38- | sepsis | 0.010140404 | 0.0072561 | 0.175048 | 33.07275382 | 25 | 0.12925918 |
| GCST90001498 | Basophil %CD33dim HLA DR- CD66b- | sepsis | -0.006754606 | 0.0093688 | 0.480729 | 24.46320231 | 18 | 0.14043054 |
| GCST90001600 | CD28+ DN (CD4-CD8-) %T cell | sepsis | -0.003836741 | 0.0255867 | 0.883785 | 8.481266288 | 11 | 0.66965358 |
| GCST90001509 | CD20 on naive-mature B cell | sepsis | -0.002722144 | 0.0059744 | 0.652433 | 21.20387668 | 27 | 0.77664741 |
| GCST90001934 | CD3 on CD8br | sepsis | 0.004782605 | 0.0056789 | 0.409654 | 10.82725546 | 21 | 0.96604613 |
| GCST90001565 | CD33 on CD66b++ myeloid cell | sepsis | 0.002644748 | 0.0071174 | 0.716183 | 14.68627373 | 14 | 0.399931 |
| GCST90001857 | CD14- CD16+ monocyte %monocyte | sepsis | 0.004385194 | 0.0073928 | 0.560061 | 12.60383942 | 20 | 0.8937275 |
| GCST90001909 | CD14+ CD16+ monocyte %monocyte | sepsis | -0.016640529 | 0.0085845 | 0.073002 | 19.08053446 | 15 | 0.21010763 |
| GCST90001735 | IgD- CD38- %lymphocyte | sepsis | -0.00990309 | 0.0070387 | 0.172813 | 23.01309302 | 24 | 0.51902527 |
| GCST90001440 | Secreting Treg % CD4 Treg | sepsis | 0.004597288 | 0.0061993 | 0.466958 | 15.76931239 | 21 | 0.78246927 |

All statistical tests were two-sided. P< 0.05 was considered significant.

Abbreviations: SE, standard error; UVMR, univariable Mendelian randomization.

**Supplementary Table 2. UVMR directional pleiotropy test and heterogeneity test for the causal associations of immunophenotypes and sepsis.**

| **Exposure** | **Traits** | **Outcome** | **Directional pleiotropy test** | | | **Heterogeneity test** | | |
| --- | --- | --- | --- | --- | --- | --- | --- | --- |
|  |  |  | **Egger intercept** | **SE** | **P value** | **Q statistic** | **Q df** | **Q p value** |
| GCST90001394 | BAFF-R on sw mem | sepsis-critical care | 0.011154798 | 0.018161 | 0.5456665 | 24.13703522 | 22 | 0.34008746 |
| GCST90001397 | IgD on unsw mem | sepsis-critical care | -0.022965431 | 0.017743 | 0.2061145 | 23.83522302 | 29 | 0.7370139 |
| GCST90001415 | FSC-A on HLA DR+ NK | sepsis-critical care | -0.011968669 | 0.019907 | 0.5561174 | 8.677478654 | 17 | 0.94983985 |
| GCST90001416 | CD33dim HLA DR- AC | sepsis-critical care | -0.0092268 | 0.020199 | 0.6532843 | 22.32313073 | 19 | 0.26847091 |
| GCST90001447 | DN (CD4-CD8-) NKT %lymphocyte | sepsis-critical care | -0.029879977 | 0.026032 | 0.262351 | 32.03820498 | 25 | 0.15690972 |
| GCST90001479 | CD80 on granulocyte | sepsis-critical care | 0.002291876 | 0.020528 | 0.9126906 | 7.886850508 | 15 | 0.92823878 |
| GCST90001485 | CD39+ secreting Treg AC | sepsis-critical care | -0.000103586 | 0.012367 | 0.9933745 | 22.58494993 | 30 | 0.83193069 |
| GCST90001502 | CD3 on CD28+ DN (CD4-CD8-) | sepsis-critical care | -0.005357979 | 0.01526 | 0.7276633 | 37.04220769 | 35 | 0.37489788 |
| GCST90001541 | CD28+ DN (CD4-CD8-) AC | sepsis-critical care | 0.000136725 | 0.01671 | 0.9935208 | 33.57063377 | 34 | 0.48853111 |
| GCST90001556 | SSC-A on monocyte | sepsis-critical care | 0.009733602 | 0.023515 | 0.6868755 | 11.2415751 | 12 | 0.50833703 |
| GCST90001611 | CD24 on transitional | sepsis-critical care | 0.005295046 | 0.029351 | 0.8592493 | 13.89222274 | 16 | 0.60674283 |
| GCST90001624 | CD8dim %T cell | sepsis-critical care | 0.00902423 | 0.018153 | 0.623817 | 19.36160266 | 24 | 0.73244018 |
| GCST90001651 | CD24 on IgD+ CD38br | sepsis-critical care | 0.033713578 | 0.017465 | 0.0665608 | 21.1209833 | 23 | 0.57369929 |
| GCST90001660 | SSC-A on HLA DR+ T cell | sepsis-critical care | -0.009938669 | 0.017496 | 0.5748693 | 16.02056268 | 27 | 0.95255421 |
| GCST90001693 | CM CD8br %CD8br | sepsis-critical care | 0.000119957 | 0.014792 | 0.9935914 | 27.87649 | 27 | 0.41736256 |
| GCST90001729 | CCR2 on CD62L+ myeloid DC | sepsis-critical care | -0.0334771 | 0.022101 | 0.1463002 | 15.91527259 | 20 | 0.7218665 |
| GCST90001768 | CD127 on CD28+ CD45RA- CD8br | sepsis-critical care | -0.002401392 | 0.019257 | 0.9019431 | 17.98329481 | 22 | 0.70697831 |
| GCST90001774 | CM DN (CD4-CD8-) AC | sepsis-critical care | -0.029345385 | 0.026446 | 0.2825988 | 14.22059024 | 18 | 0.71460216 |
| GCST90001798 | CD66b on Gr MDSC | sepsis-critical care | -0.003002397 | 0.023256 | 0.8982013 | 32.05580546 | 29 | 0.31742911 |
| GCST90001802 | IgD- CD38br %lymphocyte | sepsis-critical care | -0.05081742 | 0.019363 | 0.0135211 | 37.46060318 | 31 | 0.19683511 |
| GCST90001808 | CD86 on granulocyte | sepsis-critical care | -0.020067 | 0.026572 | 0.4564397 | 31.66922298 | 29 | 0.33457827 |
| GCST90001849 | Activated Treg AC | sepsis-critical care | -0.007576219 | 0.022211 | 0.7359914 | 18.63395366 | 25 | 0.81432565 |
| GCST90001895 | CD127 on CD28+ CD45RA+ CD8br | sepsis-critical care | -0.036487839 | 0.134421 | 0.8312587 | 0.122275259 | 2 | 0.94069376 |
| GCST90001909 | CD14+ CD16+ monocyte %monocyte | sepsis-critical care | -0.019184999 | 0.023771 | 0.4331218 | 13.97823901 | 15 | 0.52718018 |
| GCST90001921 | HLA DR on HLA DR+ CD4+ | sepsis-critical care | 0.018779575 | 0.018155 | 0.3173426 | 7.44538157 | 16 | 0.96369188 |
| GCST90001941 | CD45 on Im MDSC | sepsis-critical care | -0.008728821 | 0.022118 | 0.7000325 | 6.80048828 | 13 | 0.91213821 |
| GCST90001984 | CD25 on IgD+ | sepsis-critical care | 0.010120705 | 0.027617 | 0.7188177 | 12.49982297 | 17 | 0.76897267 |
| GCST90001992 | CD28- CD127- CD25++ CD8br %T cell | sepsis-critical care | -0.009999479 | 0.014739 | 0.5019447 | 29.66807836 | 36 | 0.76276226 |
| GCST90002015 | Activated Treg %CD4 | sepsis-critical care | -0.002339925 | 0.019041 | 0.9037237 | 8.434035939 | 17 | 0.95636468 |
| GCST90002016 | CD8 on CD28- CD8br | sepsis-critical care | -0.003213093 | 0.01897 | 0.8674993 | 12.20671427 | 18 | 0.83638339 |
| GCST90002029 | CD19 on transitional | sepsis-critical care | -0.009027381 | 0.025852 | 0.7302599 | 17.68906435 | 23 | 0.77412993 |
| GCST90002035 | CD45RA- CD28- CD8br AC | sepsis-critical care | 0.033159599 | 0.018853 | 0.0913539 | 18.90533214 | 25 | 0.80163692 |
| GCST90002040 | CD19 on IgD- CD24- | sepsis-critical care | 0.001164837 | 0.017544 | 0.9475173 | 30.22506374 | 30 | 0.45417104 |
| GCST90002080 | CD39+ CD4+ AC | sepsis-critical care | -0.022361179 | 0.018768 | 0.2481591 | 19.97646393 | 20 | 0.45940288 |
| GCST90002086 | CD45 on T cell | sepsis-critical care | -0.003209464 | 0.020493 | 0.8771223 | 18.19656844 | 21 | 0.63653311 |
| GCST90002092 | CD28+ CD45RA- CD8dim %CD8dim | sepsis-critical care | 0.159143929 | 0.273304 | 0.6643101 | 0.486649437 | 2 | 0.78401689 |

All statistical tests were two-sided. P< 0.05 was considered significant.

Abbreviations: SE, standard error; UVMR, univariable Mendelian randomization.

**Supplementary Table 3. UVMR directional pleiotropy test and heterogeneity test for the causal associations of immunophenotypes and sepsis.**

| **Exposure** | **Traits** | **Outcome** | **Directional pleiotropy test** | | | **Heterogeneity test** | | |
| --- | --- | --- | --- | --- | --- | --- | --- | --- |
|  |  |  | **Egger intercept** | **SE** | **P value** | **Q statistic** | **Q df** | **Q p value** |
| GCST90001407 | SSC-A on myeloid DC | sepsis-28-day death | 0.009753014 | 0.017699 | 0.5902834 | 16.06271393 | 15 | 0.37789594 |
| GCST90001409 | BAFF-R on B cell | sepsis-28-day death | -0.000938083 | 0.014597 | 0.9493951 | 23.69036987 | 21 | 0.30830155 |
| GCST90001411 | SSC-A on granulocyte | sepsis-28-day death | 0.000107437 | 0.023497 | 0.9963994 | 16.60015583 | 20 | 0.6787636 |
| GCST90001416 | CD33dim HLA DR- AC | sepsis-28-day death | 0.011577356 | 0.016155 | 0.4827813 | 20.07206233 | 19 | 0.39025365 |
| GCST90001535 | CD28 on CD28+ DN (CD4-CD8-) | sepsis-28-day death | 0.009074184 | 0.014192 | 0.5279557 | 30.34229382 | 28 | 0.34704483 |
| GCST90001541 | CD28+ DN (CD4-CD8-) AC | sepsis-28-day death | -0.006814071 | 0.014061 | 0.6311471 | 25.2443425 | 34 | 0.86144539 |
| GCST90001544 | CD39+ resting Treg %resting Treg | sepsis-28-day death | 0.003364656 | 0.018033 | 0.8538672 | 11.95304343 | 21 | 0.94087681 |
| GCST90001600 | CD28+ DN (CD4-CD8-) %T cell | sepsis-28-day death | 0.018668851 | 0.062091 | 0.7698254 | 5.851443433 | 11 | 0.8830901 |
| GCST90001612 | SSC-A on CD14+ monocyte | sepsis-28-day death | 0.002424545 | 0.020147 | 0.9059203 | 11.67510843 | 15 | 0.70343566 |
| GCST90001620 | CD62L- plasmacytoid DC %DC | sepsis-28-day death | -0.00301538 | 0.016243 | 0.8541651 | 28.24558168 | 27 | 0.39839622 |
| GCST90001624 | CD8dim %T cell | sepsis-28-day death | -0.022186652 | 0.015474 | 0.1650763 | 22.73940469 | 24 | 0.53522214 |
| GCST90001684 | CCR2 on plasmacytoid DC | sepsis-28-day death | -0.029995529 | 0.028689 | 0.3134954 | 18.82697793 | 15 | 0.22168176 |
| GCST90001702 | CD45 on Mo MDSC | sepsis-28-day death | 0.004746118 | 0.024247 | 0.8471356 | 26.43246778 | 18 | 0.09027303 |
| GCST90001704 | CD28 on resting Treg | sepsis-28-day death | 0.011825191 | 0.015211 | 0.4460207 | 18.24307454 | 21 | 0.63357413 |
| GCST90001709 | CD20 on transitional | sepsis-28-day death | 0.005465524 | 0.01536 | 0.7252066 | 22.09237359 | 24 | 0.57375325 |
| GCST90001710 | CD28 on CD28+ CD45RA+ CD8br | sepsis-28-day death | 0.034774446 | 0.015416 | 0.0348786 | 23.78463252 | 22 | 0.35861906 |
| GCST90001711 | CD28- DN (CD4-CD8-) AC | sepsis-28-day death | 0.009988703 | 0.018259 | 0.5914571 | 16.80304577 | 18 | 0.53668328 |
| GCST90001712 | CD28- CD25++ CD8br %T cell | sepsis-28-day death | -0.012393521 | 0.022401 | 0.5882464 | 17.12413807 | 16 | 0.3776085 |
| GCST90001713 | CD80 on plasmacytoid DC | sepsis-28-day death | 0.027376139 | 0.025872 | 0.3092508 | 12.53923027 | 14 | 0.56308173 |
| GCST90001715 | CD39+ secreting Treg %CD4 Treg | sepsis-28-day death | 0.001283391 | 0.022374 | 0.9550145 | 19.55317412 | 16 | 0.2410228 |
| GCST90001716 | CD25 on IgD- CD38br | sepsis-28-day death | -0.006479321 | 0.01918 | 0.7390163 | 25.15643709 | 21 | 0.24045226 |
| GCST90001717 | CD25 on IgD+ CD38- | sepsis-28-day death | -0.005435256 | 0.016918 | 0.7511754 | 21.58126647 | 22 | 0.48510643 |
| GCST90001718 | SSC-A on B cell | sepsis-28-day death | 0.003552477 | 0.024711 | 0.8872847 | 25.48900388 | 19 | 0.14506775 |
| GCST90001725 | CD45 on CD66b++ myelod cell | sepsis-28-day death | -0.004273118 | 0.013908 | 0.76131 | 20.72490027 | 25 | 0.70784446 |
| GCST90001727 | CD20 on IgD+ CD38dim | sepsis-28-day death | 0.015490836 | 0.015354 | 0.3271704 | 20.50988201 | 18 | 0.30485675 |
| GCST90001730 | CD27 on IgD+ CD24+ | sepsis-28-day death | -0.004681299 | 0.014997 | 0.7577343 | 24.72364666 | 24 | 0.42090033 |
| GCST90001737 | CD66b on CD66b++ myeloid cell | sepsis-28-day death | 0.016689624 | 0.015018 | 0.2766129 | 30.9722753 | 27 | 0.2722785 |
| GCST90001739 | CCR2 on CD14+ CD16- monocyte | sepsis-28-day death | 0.002669797 | 0.021376 | 0.9017951 | 19.51727319 | 22 | 0.61320708 |
| GCST90001741 | CD28- DN (CD4-CD8-) %DN | sepsis-28-day death | -0.008701605 | 0.01376 | 0.5324481 | 20.99618016 | 28 | 0.82550827 |
| GCST90001829 | CD11c on granulocyte | sepsis-28-day death | -0.006970506 | 0.019618 | 0.7262677 | 25.11616266 | 20 | 0.1970231 |
| GCST90001866 | CD4 on CD39+ resting Treg | sepsis-28-day death | -0.012583156 | 0.030655 | 0.6886871 | 12.71272884 | 13 | 0.47023818 |
| GCST90001888 | IgD+ CD38br %lymphocyte | sepsis-28-day death | 0.032976099 | 0.014961 | 0.0388065 | 18.63606658 | 22 | 0.66768072 |
| GCST90001909 | CD14+ CD16+ monocyte %monocyte | sepsis-28-day death | -0.016778048 | 0.020203 | 0.4202148 | 9.904075115 | 15 | 0.82573006 |
| GCST90001935 | EM DN (CD4-CD8-) %T cell | sepsis-28-day death | -0.01204246 | 0.024116 | 0.6252828 | 13.86326979 | 15 | 0.53592338 |
| GCST90001974 | IgD on IgD+ CD24- | sepsis-28-day death | 0.014605763 | 0.017604 | 0.4189146 | 13.99104906 | 17 | 0.66773544 |
| GCST90001978 | HLA DR on HLA DR+ NK | sepsis-28-day death | -0.006164732 | 0.022557 | 0.7886138 | 18.87356749 | 15 | 0.21952048 |
| GCST90001984 | CD25 on IgD+ | sepsis-28-day death | 0.041302635 | 0.024283 | 0.108311 | 20.16242479 | 17 | 0.26600596 |
| GCST90002048 | CD24+ CD27+ %B cell | sepsis-28-day death | 0.010830413 | 0.02488 | 0.6704749 | 12.80235531 | 14 | 0.54214211 |
| GCST90002075 | Transitional %lymphocyte | sepsis-28-day death | -0.008165798 | 0.036722 | 0.8285019 | 9.182949665 | 11 | 0.6050096 |
| GCST90002078 | CD19 on IgD- CD38- | sepsis-28-day death | -0.00226867 | 0.016962 | 0.8947167 | 9.446718482 | 25 | 0.99789497 |
| GCST90002101 | Activated & secreting Treg AC | sepsis-28-day death | -0.056119427 | 0.036095 | 0.1510562 | 5.982034677 | 11 | 0.87456026 |
| GCST90002102 | Naive DN (CD4-CD8-) %T cell | sepsis-28-day death | -0.011520215 | 0.018094 | 0.5301195 | 22.82262991 | 26 | 0.64296116 |
| GCST90002111 | CD20 on CD20- CD38- | sepsis-28-day death | -0.020087746 | 0.02256 | 0.3872985 | 14.65389884 | 16 | 0.55011837 |
| GCST90002116 | IgD- CD38dim %B cell | sepsis-28-day death | -0.008174684 | 0.017838 | 0.651946 | 17.03064286 | 20 | 0.650983 |
| GCST90002120 | CD4 on CD45RA+ CD4+ | sepsis-28-day death | -5.80E-05 | 0.038699 | 0.9988209 | 21.18779064 | 19 | 0.32651787 |

All statistical tests were two-sided. P< 0.05 was considered significant.

Abbreviations: SE, standard error; UVMR, univariable Mendelian randomization.

**Supplementary Table 4. Causal effects of Susceptibility of sepsis on different immune traits.**

| Trait | outcome | method | nsnp | pval | or | or_lci95 | or_uci95 | q value |
| --- | --- | --- | --- | --- | --- | --- | --- | --- |
| CD33- HLA DR+ AC | sepsis | Inverse variance weighted | 18 | 0.0002 | 0.9447 | 0.9163 | 0.9739 | 0.1175 |
| CD19 on transitional | sepsis | Inverse variance weighted | 24 | 0.0003 | 0.9272 | 0.8898 | 0.9662 | 0.1175 |
| CD80 on plasmacytoid DC | sepsis | Inverse variance weighted | 15 | 0.0010 | 0.9186 | 0.8733 | 0.9663 | 0.2199 |
| BAFF-R on IgD+ CD38br | sepsis | Inverse variance weighted | 19 | 0.0012 | 1.0508 | 1.0198 | 1.0829 | 0.2199 |
| IgD- CD27- AC | sepsis | Inverse variance weighted | 20 | 0.0018 | 1.0509 | 1.0186 | 1.0842 | 0.2664 |
| CCR2 on monocyte | sepsis | Inverse variance weighted | 29 | 0.0031 | 0.9699 | 0.9504 | 0.9898 | 0.3826 |
| HLA DR+ CD8br %T cell | sepsis | Inverse variance weighted | 21 | 0.0060 | 0.9668 | 0.9438 | 0.9904 | 0.5819 |
| BAFF-R on IgD+ CD38- | sepsis | Inverse variance weighted | 23 | 0.0070 | 0.9620 | 0.9352 | 0.9895 | 0.5819 |
| TD CD4+ %T cell | sepsis | Inverse variance weighted | 17 | 0.0072 | 0.9309 | 0.8836 | 0.9808 | 0.5819 |
| TD CD8br AC | sepsis | Inverse variance weighted | 24 | 0.0087 | 0.9525 | 0.9185 | 0.9878 | 0.6361 |
| CD4+ %T cell | sepsis | Inverse variance weighted | 20 | 0.0097 | 0.9528 | 0.9185 | 0.9883 | 0.6425 |
| CD4 on CD39+ resting Treg | sepsis | Inverse variance weighted | 13 | 0.0113 | 1.0534 | 1.0118 | 1.0967 | 0.6901 |
| SSC-A on monocyte | sepsis | Inverse variance weighted | 13 | 0.0132 | 0.9666 | 0.9409 | 0.9929 | 0.7299 |
| CD45 on CD4+ | sepsis | Inverse variance weighted | 15 | 0.0146 | 1.0465 | 1.0090 | 1.0853 | 0.7299 |
| Naive DN (CD4-CD8-) AC | sepsis | Inverse variance weighted | 25 | 0.0153 | 0.9749 | 0.9551 | 0.9951 | 0.7299 |
| CD45 on HLA DR+ T cell | sepsis | Inverse variance weighted | 19 | 0.0164 | 1.0326 | 1.0059 | 1.0601 | 0.7299 |
| CD11c+ HLA DR++ monocyte AC | sepsis | Inverse variance weighted | 22 | 0.0187 | 0.9813 | 0.9659 | 0.9969 | 0.7299 |
| EM CD8br %T cell | sepsis | Inverse variance weighted | 19 | 0.0193 | 1.0453 | 1.0072 | 1.0847 | 0.7299 |
| CD33 on CD33br HLA DR+ | sepsis | Inverse variance weighted | 21 | 0.0194 | 0.9563 | 0.9211 | 0.9928 | 0.7299 |
| CD19 on memory B cell | sepsis | Inverse variance weighted | 17 | 0.0200 | 0.9668 | 0.9396 | 0.9947 | 0.7299 |
| CD28+ CD45RA- CD8dim AC | sepsis | Inverse variance weighted | 24 | 0.0233 | 1.0320 | 1.0043 | 1.0605 | 0.8104 |
| IgD- CD38dim %lymphocyte | sepsis | Inverse variance weighted | 23 | 0.0244 | 0.9795 | 0.9621 | 0.9973 | 0.8109 |
| CD20 on B cell | sepsis | Inverse variance weighted | 19 | 0.0266 | 0.9544 | 0.9159 | 0.9946 | 0.8186 |
| CD25 on CD20- CD38- | sepsis | Inverse variance weighted | 26 | 0.0281 | 0.9805 | 0.9635 | 0.9979 | 0.8186 |
| Resting Treg AC | sepsis | Inverse variance weighted | 25 | 0.0283 | 0.9823 | 0.9668 | 0.9981 | 0.8186 |
| CD45 on lymphocyte | sepsis | Inverse variance weighted | 7 | 0.0291 | 1.0721 | 1.0071 | 1.1412 | 0.8186 |
| CD20 on IgD+ CD38- | sepsis | Inverse variance weighted | 26 | 0.0314 | 0.9587 | 0.9225 | 0.9962 | 0.8508 |
| Basophil %CD33dim HLA DR- CD66b- | sepsis | Inverse variance weighted | 19 | 0.0348 | 0.9476 | 0.9013 | 0.9962 | 0.9075 |
| CD28+ DN (CD4-CD8-) %T cell | sepsis | Inverse variance weighted | 12 | 0.0367 | 0.9526 | 0.9102 | 0.9970 | 0.9250 |
| CD20 on naive-mature B cell | sepsis | Inverse variance weighted | 28 | 0.0385 | 1.0143 | 1.0008 | 1.0280 | 0.9372 |
| CD3 on CD8br | sepsis | Inverse variance weighted | 22 | 0.0411 | 1.0297 | 1.0012 | 1.0591 | 0.9598 |
| CD33 on CD66b++ myeloid cell | sepsis | Inverse variance weighted | 15 | 0.0420 | 1.0356 | 1.0013 | 1.0711 | 0.9598 |
| CD14- CD16+ monocyte %monocyte | sepsis | Inverse variance weighted | 21 | 0.0443 | 1.0269 | 1.0007 | 1.0538 | 0.9804 |
| CD14+ CD16+ monocyte %monocyte | sepsis | Inverse variance weighted | 16 | 0.0481 | 1.0469 | 1.0004 | 1.0956 | 0.9805 |
| IgD- CD38- %lymphocyte | sepsis | Inverse variance weighted | 25 | 0.0483 | 1.0366 | 1.0003 | 1.0742 | 0.9805 |
| Secreting Treg % CD4 Treg | sepsis | Inverse variance weighted | 22 | 0.0492 | 1.0350 | 1.0001 | 1.0710 | 0.9805 |

**Supplementary Table 5. Causal effects of severity of sepsis on different immune traits.**

| Trait | outcome.x | method | nsnp | pval | or | or_lci95 | or_uci95 | p_adjusted |
| --- | --- | --- | --- | --- | --- | --- | --- | --- |
| BAFF-R on sw mem | Sepsis (critical care) | Inverse variance weighted | 23 | 0.0286 | 1.0674 | 1.0069 | 1.1315 | 0.9298 |
| IgD on unsw mem | Sepsis (critical care) | Inverse variance weighted | 30 | 0.0056 | 1.1427 | 1.0397 | 1.2559 | 0.9298 |
| FSC-A on HLA DR+ NK | Sepsis (critical care) | Inverse variance weighted | 18 | 0.0063 | 0.8791 | 0.8015 | 0.9643 | 0.9298 |
| CD33dim HLA DR- AC | Sepsis (critical care) | Inverse variance weighted | 20 | 0.0456 | 1.0698 | 1.0013 | 1.1429 | 0.9298 |
| DN (CD4-CD8-) NKT %lymphocyte | Sepsis (critical care) | Inverse variance weighted | 26 | 0.0217 | 0.9011 | 0.8244 | 0.9849 | 0.9298 |
| CD80 on granulocyte | Sepsis (critical care) | Inverse variance weighted | 16 | 0.0168 | 1.1305 | 1.0224 | 1.2500 | 0.9298 |
| CD39+ secreting Treg AC | Sepsis (critical care) | Inverse variance weighted | 31 | 0.0231 | 0.9733 | 0.9508 | 0.9963 | 0.9298 |
| CD3 on CD28+ DN (CD4-CD8-) | Sepsis (critical care) | Inverse variance weighted | 36 | 0.0363 | 1.0383 | 1.0024 | 1.0755 | 0.9298 |
| CD28+ DN (CD4-CD8-) AC | Sepsis (critical care) | Inverse variance weighted | 35 | 0.0287 | 0.9053 | 0.8281 | 0.9897 | 0.9298 |
| SSC-A on monocyte | Sepsis (critical care) | Inverse variance weighted | 13 | 0.0200 | 0.9125 | 0.8448 | 0.9857 | 0.9298 |
| CD24 on transitional | Sepsis (critical care) | Inverse variance weighted | 17 | 0.0458 | 1.1344 | 1.0024 | 1.2837 | 0.9298 |
| CD8dim %T cell | Sepsis (critical care) | Inverse variance weighted | 25 | 0.0396 | 1.0941 | 1.0043 | 1.1919 | 0.9298 |
| CD24 on IgD+ CD38br | Sepsis (critical care) | Inverse variance weighted | 24 | 0.0259 | 0.8907 | 0.8044 | 0.9862 | 0.9298 |
| SSC-A on HLA DR+ T cell | Sepsis (critical care) | Inverse variance weighted | 28 | 0.0373 | 0.9260 | 0.8614 | 0.9955 | 0.9298 |
| CM CD8br %CD8br | Sepsis (critical care) | Inverse variance weighted | 28 | 0.0437 | 1.0529 | 1.0015 | 1.1071 | 0.9298 |
| CCR2 on CD62L+ myeloid DC | Sepsis (critical care) | Inverse variance weighted | 21 | 0.0448 | 0.8976 | 0.8077 | 0.9975 | 0.9298 |
| CD127 on CD28+ CD45RA- CD8br | Sepsis (critical care) | Inverse variance weighted | 23 | 0.0385 | 1.0884 | 1.0045 | 1.1792 | 0.9298 |
| CM DN (CD4-CD8-) AC | Sepsis (critical care) | Inverse variance weighted | 19 | 0.0314 | 0.8863 | 0.7940 | 0.9893 | 0.9298 |
| CD66b on Gr MDSC | Sepsis (critical care) | Inverse variance weighted | 30 | 0.0112 | 1.1051 | 1.0230 | 1.1937 | 0.9298 |
| IgD- CD38br %lymphocyte | Sepsis (critical care) | Inverse variance weighted | 32 | 0.0352 | 1.1037 | 1.0069 | 1.2099 | 0.9298 |
| CD86 on granulocyte | Sepsis (critical care) | Inverse variance weighted | 30 | 0.0423 | 1.0949 | 1.0032 | 1.1949 | 0.9298 |
| Activated Treg AC | Sepsis (critical care) | Inverse variance weighted | 26 | 0.0362 | 0.9062 | 0.8265 | 0.9937 | 0.9298 |
| CD127 on CD28+ CD45RA+ CD8br | Sepsis (critical care) | Inverse variance weighted | 3 | 0.0170 | 1.6726 | 1.0964 | 2.5515 | 0.9298 |
| CD14+ CD16+ monocyte %monocyte | Sepsis (critical care) | Inverse variance weighted | 16 | 0.0276 | 1.1389 | 1.0145 | 1.2785 | 0.9298 |
| HLA DR on HLA DR+ CD4+ | Sepsis (critical care) | Inverse variance weighted | 17 | 0.0176 | 0.9054 | 0.8341 | 0.9828 | 0.9298 |
| CD45 on Im MDSC | Sepsis (critical care) | Inverse variance weighted | 14 | 0.0183 | 0.9172 | 0.8536 | 0.9855 | 0.9298 |
| CD25 on IgD+ | Sepsis (critical care) | Inverse variance weighted | 18 | 0.0242 | 0.8877 | 0.8004 | 0.9846 | 0.9298 |
| CD28- CD127- CD25++ CD8br %T cell | Sepsis (critical care) | Inverse variance weighted | 37 | 0.0349 | 0.9538 | 0.9128 | 0.9967 | 0.9298 |
| Activated Treg %CD4 | Sepsis (critical care) | Inverse variance weighted | 18 | 0.0183 | 0.8990 | 0.8229 | 0.9821 | 0.9298 |
| CD8 on CD28- CD8br | Sepsis (critical care) | Inverse variance weighted | 19 | 0.0285 | 0.9063 | 0.8300 | 0.9897 | 0.9298 |
| CD19 on transitional | Sepsis (critical care) | Inverse variance weighted | 24 | 0.0003 | 0.8201 | 0.7375 | 0.9120 | 0.1838 |
| CD45RA- CD28- CD8br AC | Sepsis (critical care) | Inverse variance weighted | 26 | 0.0159 | 1.1251 | 1.0223 | 1.2382 | 0.9298 |
| CD19 on IgD- CD24- | Sepsis (critical care) | Inverse variance weighted | 31 | 0.0044 | 0.8879 | 0.8181 | 0.9635 | 0.9298 |
| CD39+ CD4+ AC | Sepsis (critical care) | Inverse variance weighted | 21 | 0.0421 | 0.8877 | 0.7915 | 0.9958 | 0.9298 |
| CD45 on T cell | Sepsis (critical care) | Inverse variance weighted | 22 | 0.0437 | 0.9011 | 0.8144 | 0.9971 | 0.9298 |
| CD28+ CD45RA- CD8dim %CD8dim | Sepsis (critical care) | Inverse variance weighted | 3 | 0.0285 | 1.0930 | 1.0094 | 1.1836 | 0.9298 |

**Supplementary Table 6. Causal effects of mortality of sepsis on different immune traits.**

| Trait | outcome.x | method | nsnp | pval | or | or_lci95 | or_uci95 | p_adjusted |
| --- | --- | --- | --- | --- | --- | --- | --- | --- |
| SSC-A on myeloid DC | Sepsis (28 day death) | Inverse variance weighted | 16 | 0.035565 | 0.913655 | 0.839869 | 0.993924 | 0.697096 |
| BAFF-R on B cell | Sepsis (28 day death) | Inverse variance weighted | 22 | 0.013599 | 0.913189 | 0.849641 | 0.981491 | 0.662738 |
| SSC-A on granulocyte | Sepsis (28 day death) | Inverse variance weighted | 21 | 0.000591 | 0.811608 | 0.720495 | 0.914243 | 0.143988 |
| CD33dim HLA DR- AC | Sepsis (28 day death) | Inverse variance weighted | 20 | 0.049677 | 0.947964 | 0.898702 | 0.999926 | 0.766654 |
| CD28 on CD28+ DN (CD4-CD8-) | Sepsis (28 day death) | Inverse variance weighted | 29 | 0.023134 | 1.092415 | 1.012186 | 1.179005 | 0.697096 |
| CD28+ DN (CD4-CD8-) AC | Sepsis (28 day death) | Inverse variance weighted | 35 | 0.01328 | 0.908867 | 0.842658 | 0.980278 | 0.662738 |
| CD39+ resting Treg %resting Treg | Sepsis (28 day death) | Inverse variance weighted | 22 | 0.031971 | 1.090868 | 1.007521 | 1.181109 | 0.697096 |
| CD28+ DN (CD4-CD8-) %T cell | Sepsis (28 day death) | Inverse variance weighted | 12 | 0.006615 | 0.858061 | 0.768307 | 0.9583 | 0.549516 |
| SSC-A on CD14+ monocyte | Sepsis (28 day death) | Inverse variance weighted | 16 | 0.031568 | 0.910474 | 0.835857 | 0.991752 | 0.697096 |
| CD62L- plasmacytoid DC %DC | Sepsis (28 day death) | Inverse variance weighted | 28 | 0.012028 | 1.077666 | 1.016556 | 1.14245 | 0.662738 |
| CD8dim %T cell | Sepsis (28 day death) | Inverse variance weighted | 25 | 0.042529 | 1.078526 | 1.002549 | 1.16026 | 0.740207 |
| CCR2 on plasmacytoid DC | Sepsis (28 day death) | Inverse variance weighted | 16 | 0.047771 | 0.879314 | 0.774168 | 0.99874 | 0.766654 |
| CD45 on Mo MDSC | Sepsis (28 day death) | Inverse variance weighted | 19 | 0.02898 | 0.928582 | 0.868837 | 0.992436 | 0.697096 |
| CD28 on resting Treg | Sepsis (28 day death) | Inverse variance weighted | 22 | 0.036238 | 0.942662 | 0.891981 | 0.996222 | 0.697096 |
| CD20 on transitional | Sepsis (28 day death) | Inverse variance weighted | 25 | 0.034425 | 0.94943 | 0.904853 | 0.996203 | 0.697096 |
| CD28 on CD28+ CD45RA+ CD8br | Sepsis (28 day death) | Inverse variance weighted | 23 | 0.020344 | 0.927366 | 0.870129 | 0.988369 | 0.697096 |
| CD28- DN (CD4-CD8-) AC | Sepsis (28 day death) | Inverse variance weighted | 19 | 0.00915 | 0.927827 | 0.877006 | 0.981593 | 0.574811 |
| CD28- CD25++ CD8br %T cell | Sepsis (28 day death) | Inverse variance weighted | 17 | 0.001188 | 0.910874 | 0.860886 | 0.963765 | 0.21718 |
| CD80 on plasmacytoid DC | Sepsis (28 day death) | Inverse variance weighted | 15 | 0.000159 | 0.788553 | 0.697072 | 0.892038 | 0.058265 |
| CD39+ secreting Treg %CD4 Treg | Sepsis (28 day death) | Inverse variance weighted | 17 | 0.007642 | 0.916221 | 0.85917 | 0.97706 | 0.558626 |
| CD25 on IgD- CD38br | Sepsis (28 day death) | Inverse variance weighted | 22 | 0.025445 | 0.932426 | 0.876925 | 0.99144 | 0.697096 |
| CD25 on IgD+ CD38- | Sepsis (28 day death) | Inverse variance weighted | 23 | 0.001548 | 0.919793 | 0.873388 | 0.968663 | 0.22639 |
| SSC-A on B cell | Sepsis (28 day death) | Inverse variance weighted | 20 | 0.019781 | 0.927545 | 0.870688 | 0.988114 | 0.697096 |
| CD45 on CD66b++ myelod cell | Sepsis (28 day death) | Inverse variance weighted | 26 | 0.017382 | 1.071182 | 1.012173 | 1.13363 | 0.668737 |
| CD20 on IgD+ CD38dim | Sepsis (28 day death) | Inverse variance weighted | 19 | 0.034331 | 1.07376 | 1.005266 | 1.146921 | 0.697096 |
| CD27 on IgD+ CD24+ | Sepsis (28 day death) | Inverse variance weighted | 25 | 0.016053 | 1.071154 | 1.012864 | 1.132798 | 0.668737 |
| CD66b on CD66b++ myeloid cell | Sepsis (28 day death) | Inverse variance weighted | 28 | 0.027323 | 1.075946 | 1.008222 | 1.14822 | 0.697096 |
| CCR2 on CD14+ CD16- monocyte | Sepsis (28 day death) | Inverse variance weighted | 23 | 0.03986 | 1.115595 | 1.00508 | 1.238261 | 0.71851 |
| CD28- DN (CD4-CD8-) %DN | Sepsis (28 day death) | Inverse variance weighted | 29 | 0.035682 | 1.061883 | 1.004025 | 1.123075 | 0.697096 |
| CD11c on granulocyte | Sepsis (28 day death) | Inverse variance weighted | 21 | 0.028631 | 0.931184 | 0.873583 | 0.992583 | 0.697096 |
| CD4 on CD39+ resting Treg | Sepsis (28 day death) | Inverse variance weighted | 14 | 0.040299 | 1.106078 | 1.004468 | 1.217966 | 0.71851 |
| IgD+ CD38br %lymphocyte | Sepsis (28 day death) | Inverse variance weighted | 23 | 0.006766 | 1.101952 | 1.027183 | 1.182163 | 0.549516 |
| CD14+ CD16+ monocyte %monocyte | Sepsis (28 day death) | Inverse variance weighted | 16 | 8.31E-05 | 1.218157 | 1.104127 | 1.343964 | 0.058265 |
| EM DN (CD4-CD8-) %T cell | Sepsis (28 day death) | Inverse variance weighted | 16 | 0.005135 | 1.169708 | 1.048086 | 1.305444 | 0.536223 |
| IgD on IgD+ CD24- | Sepsis (28 day death) | Inverse variance weighted | 18 | 0.032102 | 0.884978 | 0.791407 | 0.989612 | 0.697096 |
| HLA DR on HLA DR+ NK | Sepsis (28 day death) | Inverse variance weighted | 16 | 0.003227 | 1.105651 | 1.034167 | 1.182075 | 0.393192 |
| CD25 on IgD+ | Sepsis (28 day death) | Inverse variance weighted | 18 | 0.039632 | 0.904144 | 0.821391 | 0.995233 | 0.71851 |
| CD24+ CD27+ %B cell | Sepsis (28 day death) | Inverse variance weighted | 15 | 0.032172 | 0.90936 | 0.833647 | 0.991949 | 0.697096 |
| Transitional %lymphocyte | Sepsis (28 day death) | Inverse variance weighted | 12 | 0.021675 | 0.844298 | 0.730716 | 0.975535 | 0.697096 |
| CD19 on IgD- CD38- | Sepsis (28 day death) | Inverse variance weighted | 26 | 0.044225 | 0.929193 | 0.865039 | 0.998106 | 0.751827 |
| Activated & secreting Treg AC | Sepsis (28 day death) | Inverse variance weighted | 12 | 0.028843 | 1.1687 | 1.016223 | 1.344056 | 0.697096 |
| Naive DN (CD4-CD8-) %T cell | Sepsis (28 day death) | Inverse variance weighted | 27 | 0.009436 | 0.948202 | 0.910877 | 0.987056 | 0.574811 |
| CD20 on CD20- CD38- | Sepsis (28 day death) | Inverse variance weighted | 17 | 0.014717 | 0.915596 | 0.852967 | 0.982824 | 0.668737 |
| IgD- CD38dim %B cell | Sepsis (28 day death) | Inverse variance weighted | 21 | 0.030981 | 1.072067 | 1.006385 | 1.142035 | 0.697096 |
| CD4 on CD45RA+ CD4+ | Sepsis (28 day death) | Inverse variance weighted | 20 | 0.017294 | 0.864263 | 0.766445 | 0.974566 | 0.668737 |

**Supplementary Figure**

**Supplementary Figure 1. Mendelian randomization associations of septic outcomes on immune traits derived from the IVW analysis.**

**
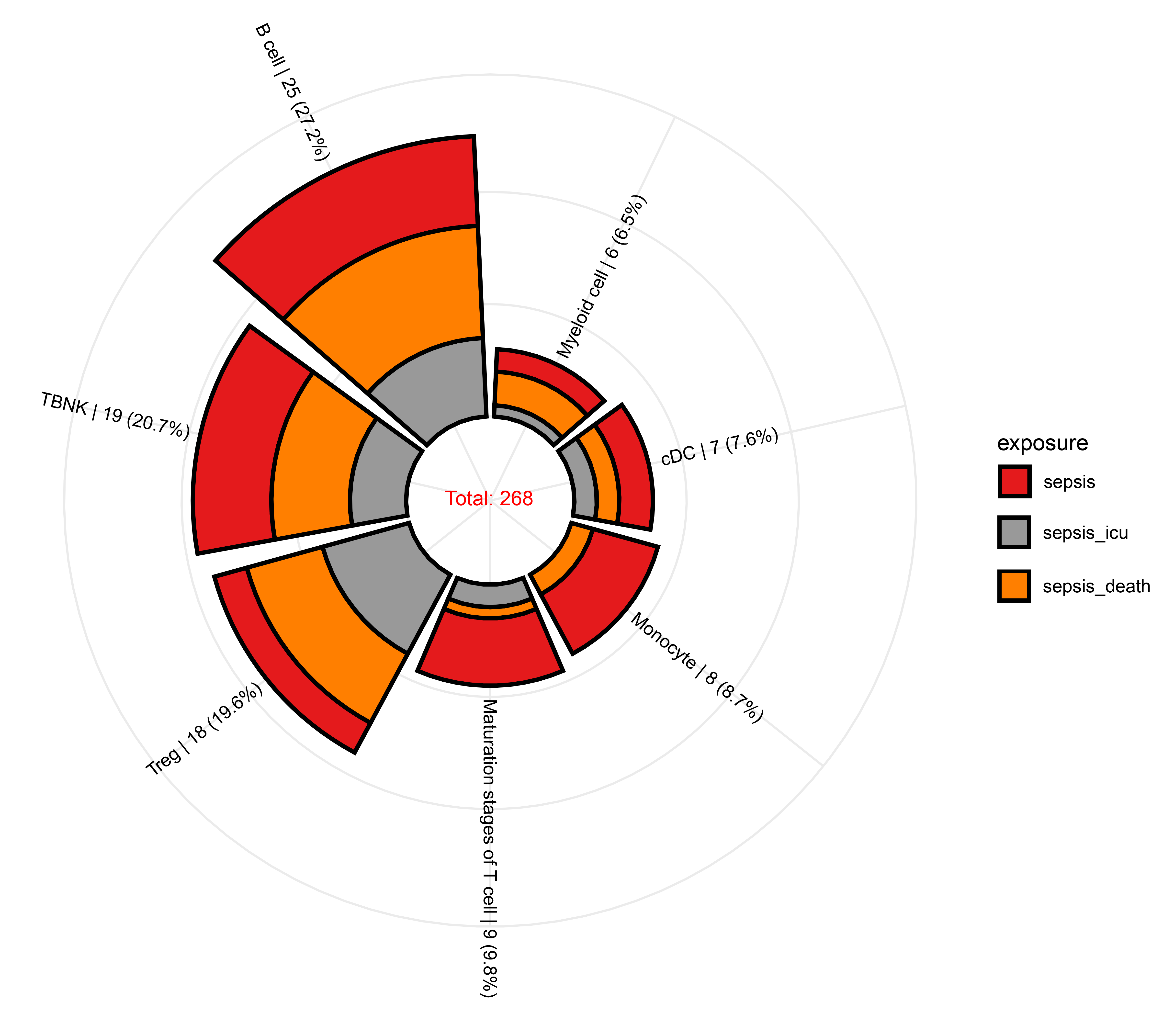
**

**Supplementary Figure 2. Forest plot of causal effects of sepsis (Susceptibility, Severity and mortality) on immunophenotypes.**

**
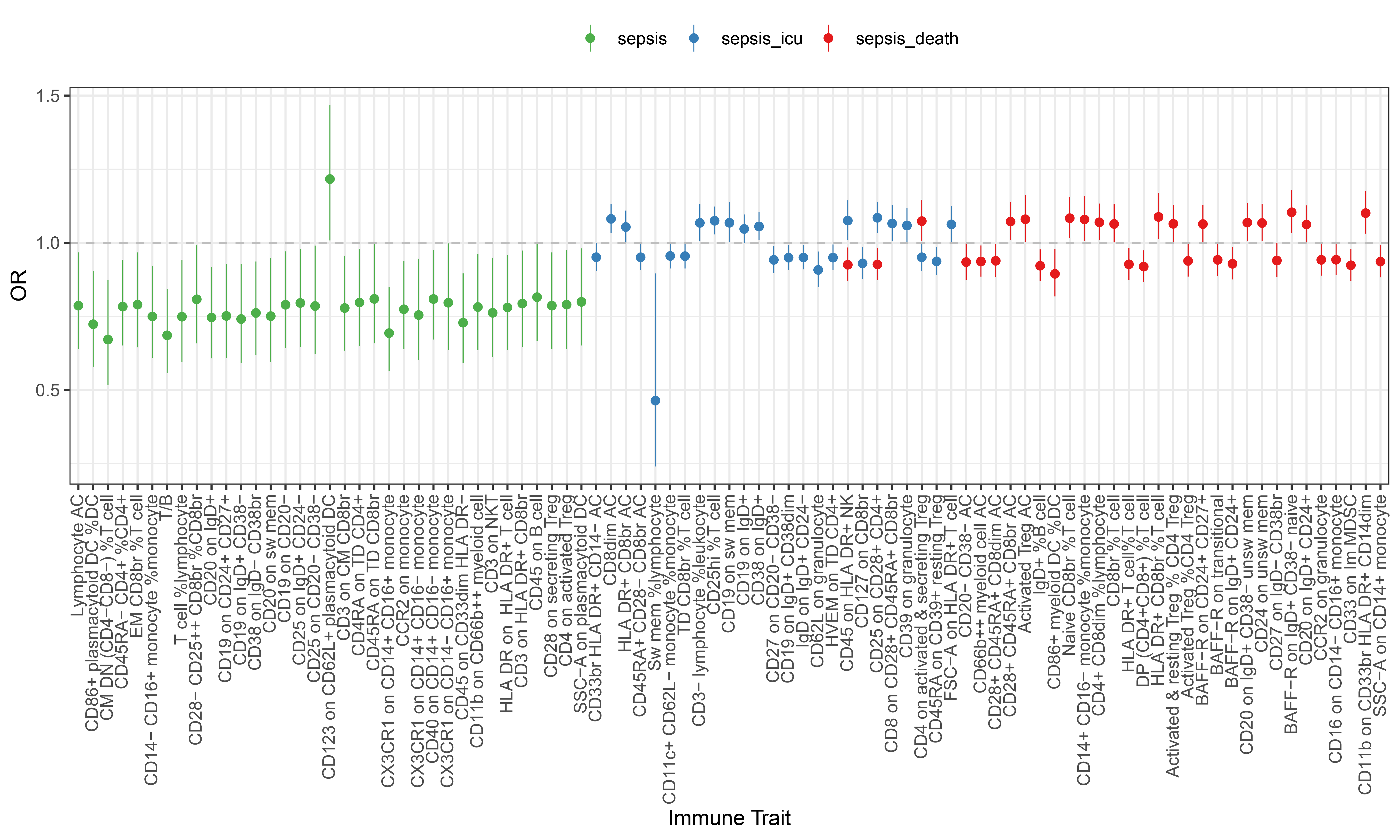
**

**Supplementary Figure 3.** **Forest plot of causal effects of different immune traits on Susceptibility of sepsis.**

**
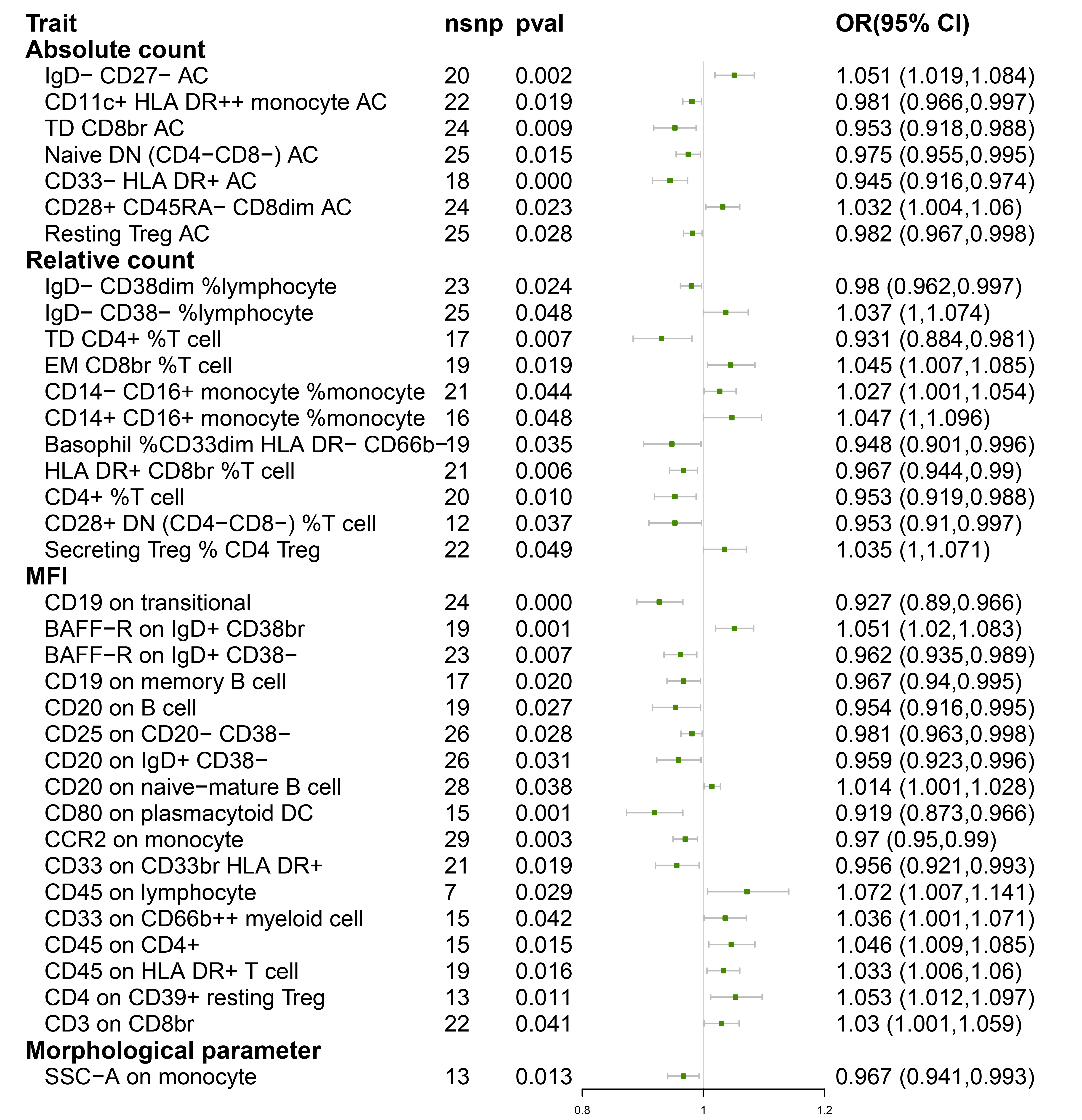
**

**Supplementary Figure 4. Forest plot of causal effects of different immune traits on Severity of sepsis.**

**
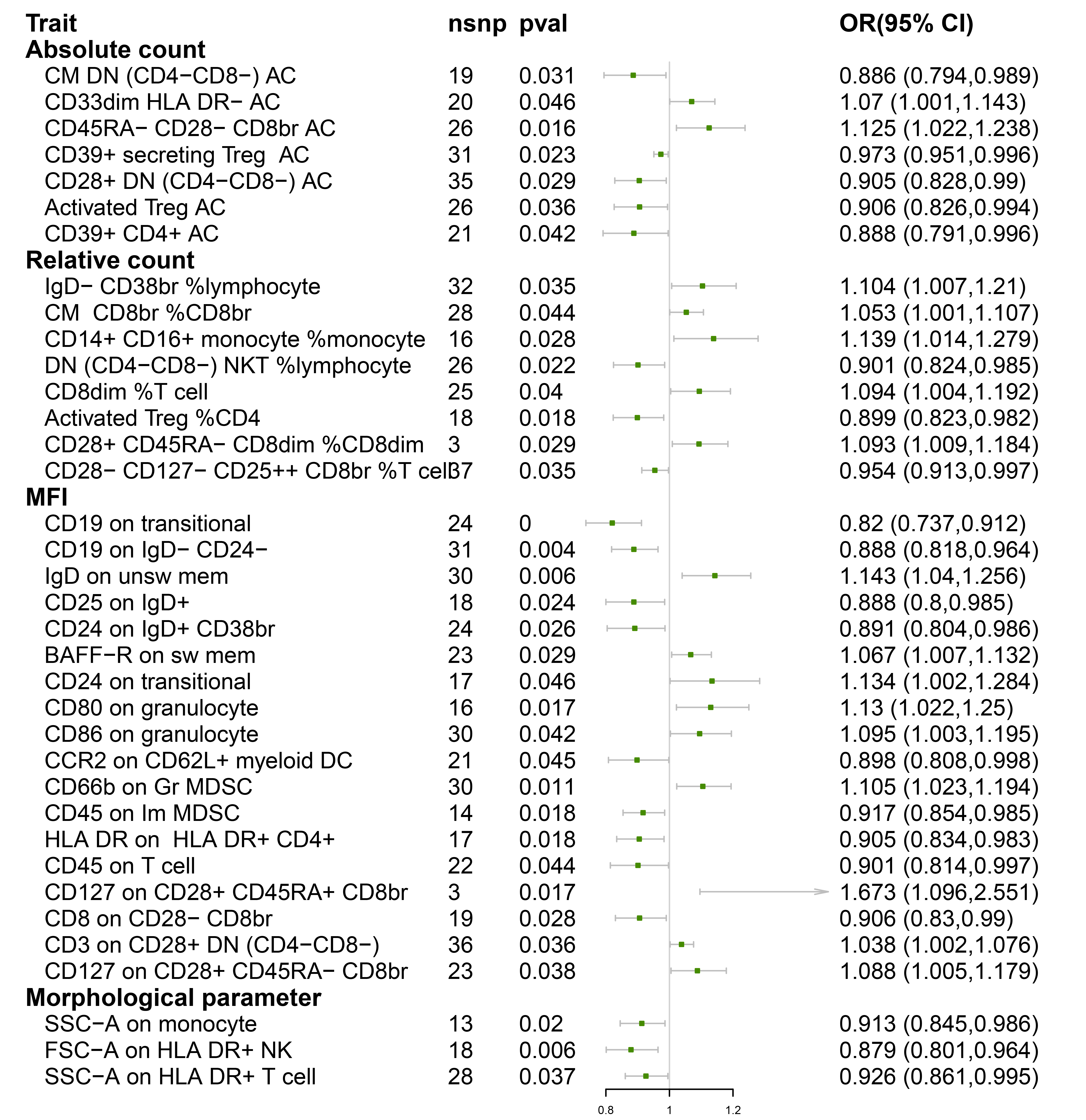
**

**Supplementary Figure 5. Forest plot of causal effects of different immune traits on mortality of sepsis.**

**
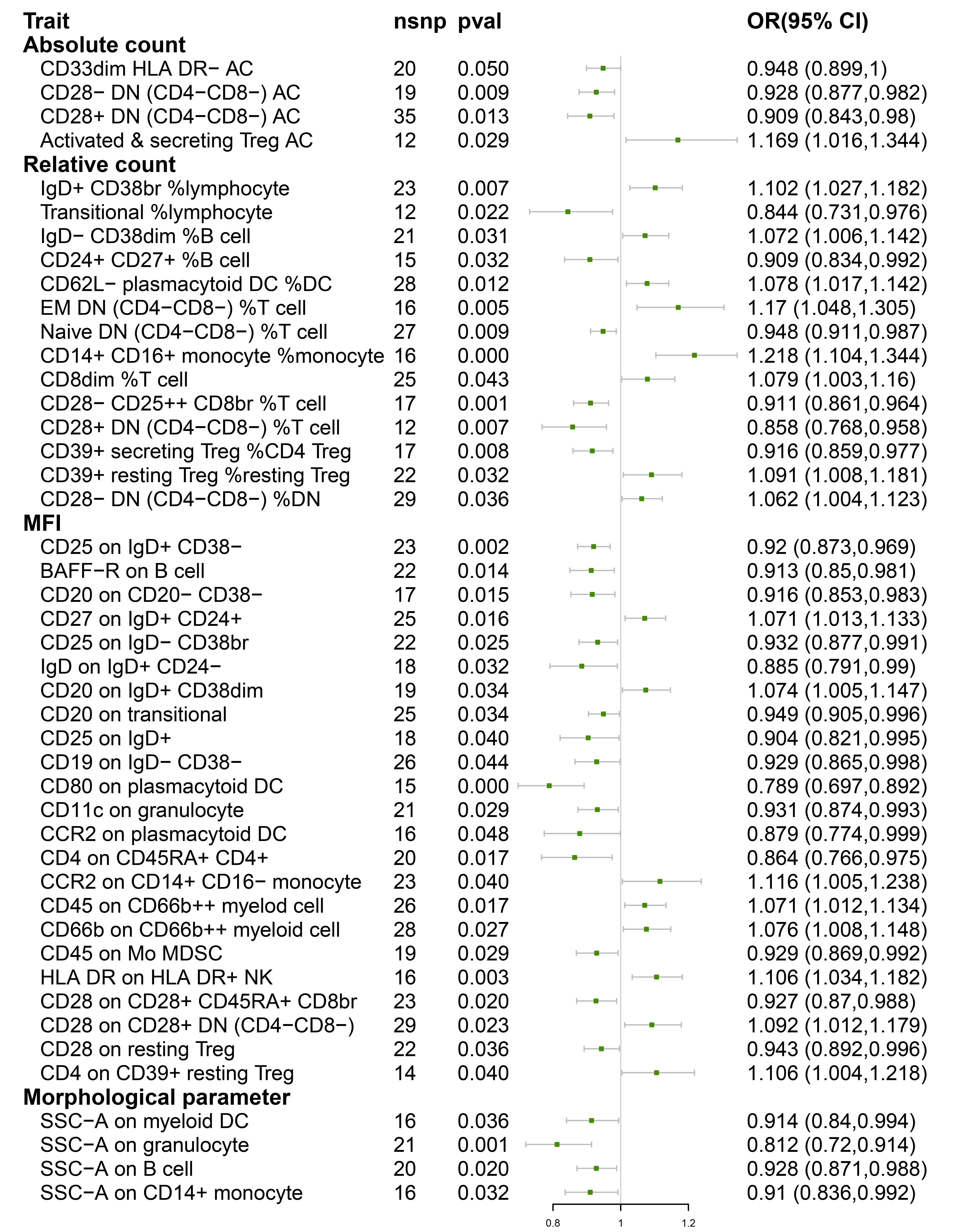
**

**Supplementary Figure Legend**

**Supplementary Figure 1. Mendelian randomization associations of septic outcomes on immune traits derived from the IVW analysis.**

Mendelian randomization associations of septic outcomes on immunophenotypes (seven panels) that derived from the IVW analysis. Immune traits positively associated with septic outcomes are shown in rose diagram.

**Supplementary Figure 2. Forest plot of causal effects of sepsis (Susceptibility, Severity and mortality) on immunophenotypes.**

Two-step MR was used to evaluate the mediating role of each mediator in the causal associations of septic outcomes on immunophenotypes. MR estimates were derived from the IVW method in UVMR. All statistical tests were two-sided. P < 0.05 was considered significant.

**Supplementary Figure 3. Forest plot of causal effects of different immune traits on Susceptibility of sepsis.**

Two-step MR was used to evaluate the mediating role of each mediator in the causal associations of immunophenotypes on susceptibility of sepsis. MR estimates were derived from the IVW method in UVMR. All statistical tests were two-sided. P < 0.05 was considered significant.

**Supplementary Figure 4. Forest plot of causal effects of different immune traits on Severity of sepsis.**

Two-step MR was used to evaluate the mediating role of each mediator in the causal associations of immunophenotypes on severity of sepsis. MR estimates were derived from the IVW method in UVMR. All statistical tests were two-sided. P < 0.05 was considered significant.

**Supplementary Figure 5. Forest plot of causal effects of different immune traits on mortality of sepsis.**

Two-step MR was used to evaluate the mediating role of each mediator in the causal associations of immunophenotypes on mortality of sepsis. MR estimates were derived from the IVW method in UVMR. All statistical tests were two-sided. P < 0.05 was considered significant.
